# Timosaponin AIII Is Preferentially Cytotoxic to Tumor Cells through Inhibition of mTOR and Induction of ER Stress

**DOI:** 10.1371/journal.pone.0007283

**Published:** 2009-09-30

**Authors:** Frank W. King, Sylvia Fong, Chandi Griffin, Mark Shoemaker, Rick Staub, Yan-Ling Zhang, Isaac Cohen, Emma Shtivelman

**Affiliations:** BioNovo Inc, Emeryville, California, United States of America; INMI, Italy

## Abstract

The aqueous extract of *Anemarrhena asphodeloides* (BN108) induces apoptosis in various cancer cell lines but is significantly less cytotoxic in non-transformed cells. Chemical fractionation of BN108 showed that its cytotoxicity is associated with timosaponins, steroidal saponins of coprostane type. Timosaponin BII (TBII) is a major saponin in BN108, but it shows little cytotoxicity. A much less abundant TAIII induces cell death in tumor cells but not in normal cells, reproducing the selectivity of the total extract BN108. Glycosidase treatment, by removing the extra sugar moiety in TBII, converts it to TAIII and confers cytotoxic activity. Analysis of the mechanisms of death induced by TAIII revealed activation of two distinct pro-apoptotic pathways: first, inhibition of mTORC1 manifested in much reduced phosphorylation of mTORC1 targets; second, induction of endoplasmic reticulum stress culminating in phosphorylation of eIF2α and activation of caspase 4. These pro-apoptotic pathways are activated by TAIII selectively in tumor cells but not in normal cells. Both pathways play a causative role in TAIII cytotoxicity, as restoration of either mTOR activity or relief of ER stress alone offer only partial protection from TAIII. Inhibition of mTORC1 and induction of ER stress apparently contribute to the induction of the previously reported autophagic response in TAIII-treated cells. TAIII induced autophagy plays a protective role in TAIII induced death signaling, and failure to mount autophagic response is associated with heightened sensitivity to TAIII induced apoptosis. The multiple death-promoting and apparently tumor-selective responses to TAIII, its ability to inhibit mTORC1, and the possibility of further enhancing its cytotoxicity by pharmacological inhibition of autophagy, make TAIII an attractive candidate for development as a cancer therapeutic agent.

## Introduction

This work describes the anti-tumor activity of the aqueous extract from the plant *Anemarrhena asphodeloides* (BN108), and one of the timosaponins present in the extract, TAIII [1]. BN108 contains a number of timosaponins [1]–[3], and a variety of biological effects have been attributed to these compounds. TAIII was recently reported to induce apoptosis and protective autophagy in HeLa cells [4]. However, the mechanism through which TAIII induces cell death remains unclear.

Induction of apoptosis is a prominent mode of cytotoxic action of many chemotherapeutic drugs. Some of them induce apoptosis through a mitochondrial pathway, but some, most notably the proteasome inhibitors, induce cell death via endoplasmic reticulum (ER) stress mediated apoptotic pathway. ER stress is elicited by a wide variety of conditions including nutrient deprivation, impaired protein degradation or secretion, calcium imbalance and many others. ER stress involves specific transcriptional and translational responses that are largely controlled by three ER resident sensor proteins: IRE1, ATF6, and PERK (reviewed in [5], [6]). Activated PERK phosphorylates eukaryotic translation initiation factor eIF2α, resulting in the general inhibition of protein synthesis, but paradoxically induces a specific increase in translation of transcription factor ATF4. ATF4, in turn, induces increases in levels of several protein chaperons. Phosphorylation of eIF2α is central in the integrated stress response, named so because it is activated by diverse stressful conditions. Sustained or severe ER stress leads to activation of caspases, in particular caspase-4, followed by apoptosis [7].

ER stress was found recently in several independent studies to have an unanticipated consequence: induction of autophagy (reviewed in [8], [9]). Autophagy is a conserved cellular pathway that serves to degrade bulk cytoplasmic material ([10], [11]). It is activated in response to nutrient and energy starvation, and after treatment with some chemotherapeutic drugs. Autophagy plays a positive role in preservation of energy and nutrients, and also contributes to degradation of misfolded proteins when this function of ER is disabled due to stress. Autophagy can play a protective role in cell survival, but often serves as a mechanism of programmed cell death (reviewed in [12]).

Autophagy is inhibited in cells under normal conditions (where nutrients, ATP and growth factors are in adequate supply) by a conserved cellular pathway centered on the Ser/Thr kinase target of rapamycin (mTOR) (reviewed in [13]). mTORC1 regulates efficiency of protein translation and promotes cell growth (reviewed in [14], [15]). The two essential direct targets of mTORC1 activity are the 70 kDa ribosomal protein S6 kinase, and the eukaryotic translation initiation factor 4E binding protein 1 (4eBP1). Phosphorylation of these proteins by mTORC1 serves to activate and sustain protein translation and is used as a read-out for mTORC1 activity. Inhibition of mTORC1 has profound negative consequences for protein translation and cell growth, and at the same time activates autophagy. Another mTOR containing complex, mTORC2, promotes cell signaling through phosphorylation and activation of the pro-survival and pro-proliferative kinase Akt [16]. mTOR kinase in the mTORC2 complex is thought to be not sensitive to inhibition by rapamycin [17], [18], but inhibitors of mTOR kinase domain have been developed that suppress activity of mTOR in both mTORC complexes [19], [20]. The possible effect of mTORC2 on autophagy remains to be investigated.

This study was prompted by our observations that BN108 induced cell death selectively in a variety of cancer cell lines, but not in several immortalized non-transformed lines examined. We have therefore attempted to define the active compounds in BN108 and study their mechanism of action through examination of the mode of cell death and analysis of the effects on gene and protein expression. Timosaponin AIII was identified as a major selective cytotoxic activity in BN108, and its selective cytotoxic activity involves inhibition of mTOR, induction of ER stress and protective autophagy.

## Materials and Methods

### Reagents and antibodies

Chloroquine and EGF were purchased from Sigma. Caspase activity substrates, AnnexinV- Alexa 488 conjugate and Vibrant Lipid Raft labeling kit were purchased from Molecular Probes/InVitrogen. Caspase inhibitors were purchased from EMD Biosciences, TAIII from Wako Pure Chemical Industries, TBII and sarsasapogenin from AAPIN Chemicals, UK. Antibodies to Bim, phosphorylated and total S6 ribosomal protein, phosphoAkt S473, Akt, phospho4eBP1 and 4eBP1, phosphoeIF2α and eIF2α were from Cell Signaling Technology, ATP-citrate lyase from Epitomics, Id1 and Id3 from Biocheck; REDD1/DDIT4 and IDI1 from Protein Tech Group; SREBP-2 from BD Biosciences, INSIG-1 from Biovision, PARP from Zymed, MYC, GRP78 and GAPDH from Santa Cruz Biotechnology.

### Cell culture and treatments

All cell lines were obtained from the ATCC, and propagated according to the instructions provided. BN108 tea-like extract for use in cell culture was prepared in water as described above and freeze-dried. The resulting powder was weighed, dissolved in water to 50 mg/ml (dry weight per volume) and sterile-filtered. Timosaponins were dissolved in DMSO at 100 mM. Cells were treated with BN108 at a concentration of 0.5 mg/ml and with TAIII as indicated or with corresponding solvent (water or DMSO, labeled as “untreated” throughout the paper).

### Flow cytometry

For apoptosis determination cells were collected by trypsinization into their culture media, washed with PBS and stained with Annexin V- Alexa Fluor 488 (InVitrogen) and propidium iodide (PI), and analyzed immediately after 15 minute incubation using the CellQuest software on the FACScan (Becton Dickinson). All Annexin V-binding cells were considered to be apoptotic. Cell cycle analysis was performed with the ethanol fixed cells stained with PI in PBS at 20 µg/ml. Caspase activity assays were performed with the Vibrant FAM caspase assay kit (InVitrogen) according to the manufacturer's instructions.

### Activity Guided Isolation of Anti-cancer Compounds from BN108

BN108 extract was prepared by adding water to the ground dried herb (10∶1, volume : mass), then bringing the mixture to a boil. The herbal solution was allowed to simmer for 45–60 minutes, then suction filtered (Whatman 1 paper filter) to produce the crude tea. The crude tea was partitioned with an equal volume of ethyl acetate (repeated once) and the organic layers were recovered, combined and concentrated. Cytotoxic activity of the partitions was determined using CyQuant assay (Promega), which detected significant activity in the recovered organic layers. The concentrated ethyl acetate layers were subjected to open column chromatography on silica gel with hexane:ethyl acetate and ethyl acetate:methanol gradients. Active fractions were again determined by CyQuant assay and eluted from the silica column in the middle of the ethyl acetate:methanol gradient. LC/MS analysis of the active fractions identified a mixture of terpene sugar conjugates including timosaponin AIII, timosaponin BII, and several other uncharacterized saponins.

### LC-MS/MS Analysis of TAIII and TBII concentrations in BN108

Timosaponins were quantified using a triple quadrupole, API4000 LC/MS system (Sciex./Applied Biosystems, Foster City, CA) in negative MRM (multiple reaction monitory) mode with Collision Activated Dissociation (CAD) gas. The first quadrupole was set to select the deprotonated molecular ion [M–H]^−^ of TAIII (*m/z* 739) and TBII (*m/z* 921). The second quadrupole was used as collision chamber, and the third quadrupole to select the characteristic product ions of both compounds (*m/z* 577 for TAIII and *m/z* 759 for TBII). Peak area ratios obtained from MRM mode of the mass transitions for TAIII (*m/z* 739 → 577) and TBII (*m/z* 921 → 759) were used for quantification.

### Western blot analysis

Whole cell lysates were electrophoresed on SDS-PAGE and transferred to nitrocellulose membranes. Membranes were blotted with antibodies at recommended concentrations overnight at 4°C and the bound primary antibodies were detected using peroxidase-conjugated secondary antibodies. Blots were developed using SuperSignal enhanced chemiluminescence kit (Pierce) and imaged on Kodak Imager ISR2000.

### Timosaponin BII de-glycosylation

Laminarinase (Sigma L5272) was dissolved to a final concentration of 1 unit/ml in reaction buffer (120 mM sodium acetate pH 5.0, 60 mM NaCl and 40% DMSO) Timosaponin BII at final concentration of 1 mM was added to enzyme mix and incubated for 30 min. at 50°C. Sample was then heated for 5 min. at 90°C to deactivate the enzyme. Mass-spectrometric analysis of the digestion products was conducted on TOF LC/MS 6210, Agilent.

### Mass-spectromeric analysis of cellular proteins

MDAMB231 cells were treated with TAIII for 4, 8, and 16 hours or left untreated. Cells were disrupted in lysis buffer (100 mM TEAB pH 8.5, 150 mM NaCl and 1% Triton X-100), and 100 µg protein was denatured with 8 M urea and 100 mM TEAB pH 8.5 in the presence of 5 mM TCEP (reducing agent) for 2 hrs at room temperature. Cysteine residues were blocked with 50 mM iodoacetamide for 1 hr at room temperature in a final volume of 30 µl. Sample was diluted to 0.5 ml with 100 mM TEAB, 0.5 mM CaCl_2_ and then concentrated on an YM-3 Microcon (Millipore). Trypsin (Promega) was added and protein was digested for 12 hrs at 37°C. Proteins were then labeled with iTRAQ labeling reagents 114, 115, 116 or 117 (Applied Biosystems). The sample was mixed with iTRAQ reagent and ethanol (final 70%) and incubated for 1 hr at room temp. The reaction was quenched by adding an equal volume of water and incubation for 30 min. Sample was dried by spin-vap and resuspended in 0.1% formic acid for a final concentration of 1 mg/ml protein. Mass spectrometry analysis was performed on an Agilent 6500 Q-TOF LC/MS with Chip Cube. Identification of peptides and quantitation of iTRAQ labeling was done with SpectrumMill software (Agilent).

### Lentivirus - mediated siRNA expression and DNA transfection

For REDD1 silencing, siRNA constructs in the LKO plasmid vector were purchased from Open Biosystems/Thermo. Viruses were produced in HEK293 cells according to manufacturer's instructions and used to transduce cells, followed by selection in pre-determined concentration of puromycin. Human Grp78 cDNA in pCMV-ICIS expression plasmid was obtained from Openbiosystem. The plasmid was co-transfected with pCDNA3-Neo at 20∶1 ratio into MDA-MB231 cells, followed by selection in G418 and by limited dilution cloning of high GRP78 expressing clones.

## Results

### The aqueous extract of *Anemarrhena asphodeloides* Bunge (BN108) induces apoptotic cell death in many cancer cell lines but not in non-transformed cells

**Figure 1:**
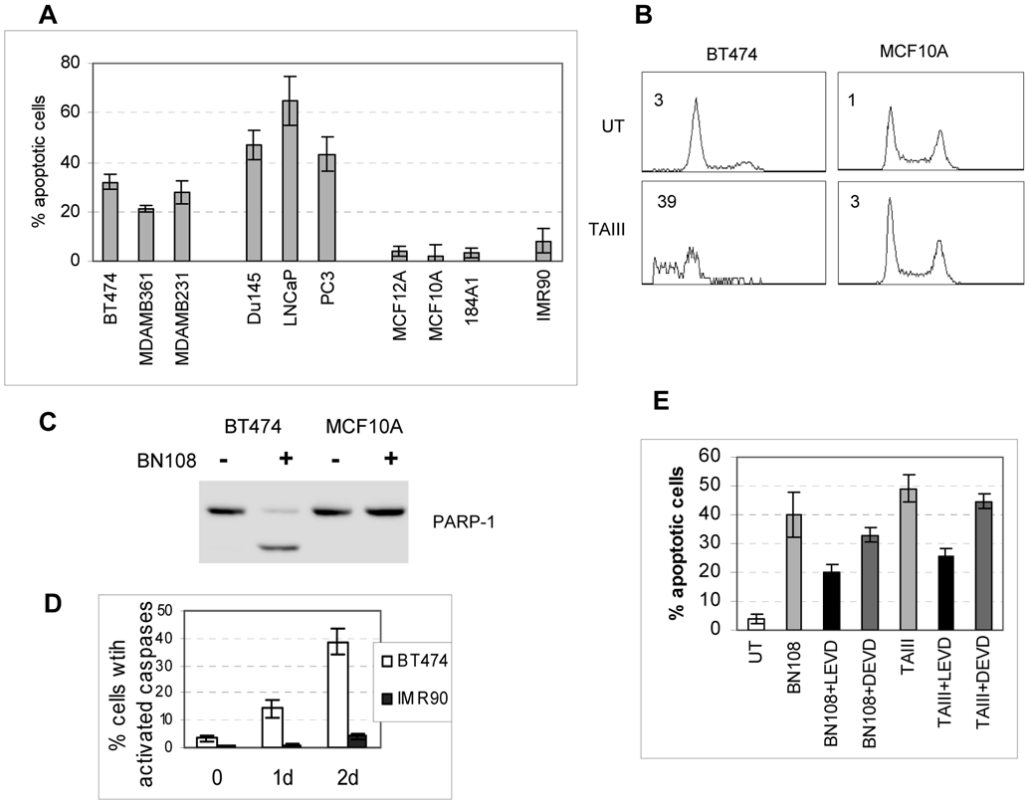
Aqueous extract from Anemarrhena asphodels induces apoptosis selectively in cancer cells. A. The indicated cell lines (three breast carcinoma lines; three prostate carcinoma lines, three immortalized mammary epithelial lines) and primary fibroblasts IMR90 were treated with 0.5 mg/ml of BN108 for 24 hours. Cells were analyzed for apoptosis by flow cytometry of Annexin V/PI stained cells. Percentage of Annexin V- binding cells is shown; results are an average of at least three experiments. B. Cell cycle analysis of BT474 and MCF10A cells treated with 0.5 mg/ml BN108 for 48 hours. Numbers show percentages of cells with DNA content less than 2n. C. Western blot analysis of PARP cleavage in BT474 and normal MCF10A cells treated with BN108 for 24 hours. D. BT474 and IMR90 cells were treated for 1 or 2 days with BN108, fixed, and cells with activated caspases were identified by flow cytometry with the Vibrant FAM caspase assay kit (InVitrogen). E. Apoptotic cells were detected in BT474 cultures as described in 1A. Cells were treated for 24 hours with BN108 (0.5 mg/ml) or TAIII (2.5 µM) with or without caspase 4 inhibitor LEVD-fmk or caspase 3 inhibitor DEVD-fmk at 10 µM.

Previous work has shown that BN108 has a strong growth inhibitory effect in a number of human cancer cell lines [21]. Several cancer and normal cell lines were analyzed for their responses to BN108 using Annexin V/propidium iodide (PI) staining to determine if BN108 induces apoptotic cell death. At the concentration of 0.5 mg/ml BN108 induced appearance of Annexin V binding cells, both PI-negative (early apoptotic) and PI positive (late apoptotic) in many of the cancer cell lines tested, but not in the non-transformed lines of mammary epithelium or in primary fibroblasts (Figure 1A). Breast cancer cell lines but not the non-transformed cells treated with BN108 also showed apoptotic DNA fragmentation (Figure 1B).

To verify the apoptotic nature of cell death induced by BN108, we have examined caspase activation in breast cancer cell line BT474 and primary fibroblasts IMR90 as well as immortalized mammary epithelium MCF10A. First, we found that proteolysis of PARP1 known to be mediated by caspases occurred in BT474 cells but not in MCF10A (Figure 1C) or IMR90 cells (not shown). Next, caspase activation in BT474 cells but not in normal cells treated by BN108 was confirmed by FACS-based assay of caspase activity (Figure 1D). Possible roles of specific caspases in apoptosis induced by BN108 were then examined using relevant inhibitors and substrates. Among different caspase inhibitors used, the caspase-4 inhibitor z-LEVD-FMK and, to a lesser degree, caspase-9 inhibitor z-LEHD-FMK, were effective in diminishing the numbers of apoptotic cells, while inhibition of caspase-3 and 6 had much less effect (Figure 1E and not shown). Direct caspase activity assays also showed specific involvement of caspases 4- and-9 (Figure S1). Involvement of caspases 4 and 9 strongly indicates that cell death induced by BN108 could involve endoplasmic reticulum stress [7].

### Structure-activity relationship between timosaponins from BN108

**Figure 2:**
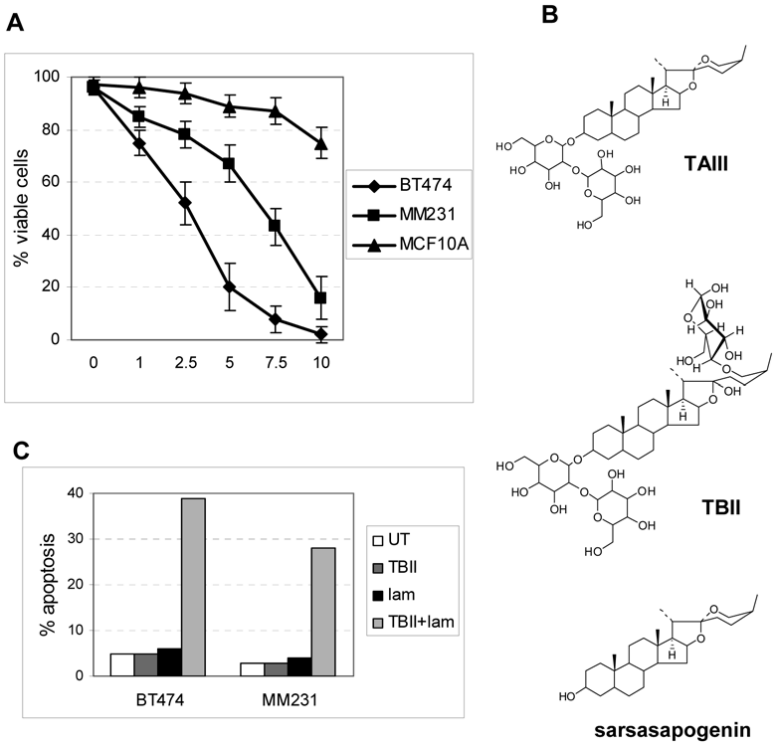
TAIII is the selectively cytotoxic compound in BN108 extract. A. Three cell lines indicated were treated with a range of concentrations of TAIII for 24 hours, and cell viability was analyzed by Annexin-PI staining. B. Structures of TAIII, TBII and sarsasapogenin. C. Cells lines were treated for 24 hours with TBII at 40 µM, heat–inactivated laminarinase alone (0.5 µg/ml), or a mixture of both after incubation for 30 min at 50°C and heat-inactivation of the enzyme. MS analysis indicated that about 20 to 30% of TBIII was converted to TAIII. Apoptotic cells were identified by Annexin V binding. The results are representative of the two separate experiments performed with very similar results.

In order to isolate the tumor-selective cytotoxic activity from BN108, we have performed chemical fractionations of the extract. Activity-guided isolation of active fractions from BN108 consistently showed that cytotoxic activity co-purifies with timosaponins, of which several were identified previously in the extracts of *Anemarrhena asphodeloides* [1], [2]. We have therefore examined if two of BN108 timosaponins, TAIII and TBII, as well as TAIII aglycone, sarsasapogenin (Figure 2A) are selectively cytotoxic to cancer cells, similarly to BN108. We have found that TAIII induced significant cell death in cancer cell lines BT474 and MDAMB231 at micromolar concentrations (Figure 2A). Non-transformed MCF10A cells were relatively resistant to TAIII at the range of concentrations tested (Figure 2A), and so were primary fibroblasts IMR90 (not shown). This selective cytotoxicity of TAIII to cancer cells recapitulates the selectivity of the total extract BN108. In addition, TAIII-induced cell death, similar to BN108, was partially suppressed in presence of caspase-4 inhibitor (Figure 1D) but was much less affected by the inhibition of caspase-3 and -6 (not shown). Timosaponin TBII and sarsasapogenin had no appreciable cytotoxic activity in any of the tested cell lines at concentrations of up to 50 µM (not shown and Figure 2C).

We have quantified the concentration of timosaponins in BN108 using LC-MS/MS. The amount of TAIII was found to be 2.15±0.09 mg/gram dry weight of the herb, and TBII 79.11±2.01 mg/g. Therefore, the concentrations of timosaponins in the culture media containing 0.5 mg/ml BN108 (routinely used in cytotoxicity assays) amount to 1.5 µM of TAIII and 37 µM of TBII in the BN108 treatment media. The concentration of TAIII in BN108 is therefore just somewhat lower than the 2.5 µM of TAIII shown to induce cell death in about 50% of treated BT474 cells (Figure 2A). This indicates that the TAIII is a major cytotoxic component of BN108, though it is possible and even likely that the extract contains additional cytotoxic substances such as other timosaponins identified previously [2].

The steroid cores of timosaponins AIII and BII are identical, but TBII contains an extra sugar moiety in addition to a shared disaccharide moiety (Figure 2B). We have therefore attempted to determine if this sugar is responsible for the lack of the cytotoxic activity of TBII. Several glycosidases were tested for their ability to cleave TBII, and laminarinase was found to convert TBII into TAIII as determined by LC/MS analysis. Laminarinase treated preparations of TBII became cytotoxic towards two breast cancer cell lines (Figure 2C) but not to MCF10A cells, similar to TAIII (not shown). We conclude that the extra sugar present in TBII interferes with its potential cytotoxicity. Enzymatic removal of this sugar moiety converts TBII to TAIII and unmasks its cytotoxicity. It will be of interest to determine if TBII could be cleaved *in vivo* by glycosidases (in particular, from intestinal microflora) to produce TAIII, thus strongly enhancing the anti-tumor activity of BN108 considering the relatively high TBII content in the extract.

### Analysis of the changes in gene expression induced by BN108 and TAIII

To try and discern the pathways involved in cell death induction by BN108, expression array analysis (Methods S1) was performed with RNAs isolated from BT474 cells prior to and after a 4 hour treatment with BN108. Only 41 transcripts were found to be upregulated more than two-fold in BT474 cells treated with BN108, and even fewer were downregulated two-fold or more (Table S1). Several genes with known anti-proliferative and/or pro-apoptotic activities were induced: REDD1/DDIT4, p21CIP, stratifin, cyclin G2, GDF15. A prominent group of BN108 induced genes represented enzymes involved in biosynthesis of cholesterol.

Among the downregulated genes with known functions were several transcripts coding for secreted proteins: amphiregulin, CYR61, thrombospondin, CXCL12, VEGF-C, as well as the pro-proliferative genes MYC and cyclin D1.

Because TAIII is at least partially responsible for the apoptosis-inducing activity of BN108, we have conducted further expression array analysis with three cell lines that showed different sensitivities to TAIII and BN108: BT474, somewhat less sensitive MDAMB231 and the relatively resistant nontransformed cell line MCF10A. TAIII treatment affected levels of many more mRNAs than BN108; but majority of transcriptional changes induced by BN108 in BT474 were also induced by TAIII. Thus, out of 42 genes upregulated by BN108 more than two-fold, 28 (i.e., two thirds) were also upregulated by TAIII. This strongly indicates that many of the cellular responses to BN108 are induced by its active constituent TAIII.

Comparisons of TAIII induced transcriptional changes between the three cell lines analyzed showed significant overlaps between BT474 and MDAMB231 (Table S2). However, the magnitudes of changes in RNA abundance were frequently different. In particular, induction of genes in the cholesterol biosynthesis pathway was significantly muted in MDAMB231 cells compared to BT474 (Table 1). On the other hand, MDAMB231 cells treated wit TAIII showed induced expression of a relatively large number of genes with functions in ER stress response and amino acid starvation, most of which were only weakly induced in BT474 (Table 2). In the non-transformed MCF10A cells the genes whose expression was significantly changed after TAIII treatment were mostly distinct from transcripts affected in cancer cell lines (Table S2), which could reflect the resistance of MCF10A to apoptosis induction by TAIII. The discernible commonalities between MCF10A transcriptional response to TAIII and that of breast cancer cell lines were a weak induction in the former of expression of some genes in the cholesterol biosynthesis pathway, ER stress response and induction of autophagy (Table 2).

**Table 1:** Partial list of genes in the cholesterol biosynthesis pathway that are upregulated in TAIII-treated cells.

		BT474	MDAMB231	MCF10A
INSIG1	insulin induced gene 1	8.71	6.14	1.84
SC4MOL	sterol-C4-methyl oxidase-like	7.64	2.07	1.35
LPIN1	lipin 1	6.69	2.31	2.22
SC4MOL	sterol-C4-methyl oxidase-like	6.10	2.08	1.24
HSD17B7	hydroxysteroid (17-beta) dehydrogenase 7	5.10	1.33	1.54
HSD17B7P2	hydroxysteroid (17-beta) dehydrogenase 7 pseudogene 2	4.73	0.98	1.10
IDI1	isopentenyl-diphosphate delta isomerase 1	4.50	1.80	1.19
MVD	mevalonate (diphospho) decarboxylase	3.95	1.20	1.39
GNE	glucosamine (UDP-N-acetyl)-2-epimerase/N-acetylmannosamine kinase	3.83	1.49	0.98
LDLR	low density lipoprotein receptor (familial hypercholesterolemia)	3.54	2.37	1.70
SQLE	squalene epoxidase	3.32	1.05	1.27
PGM2L1	phosphoglucomutase 2-like 1	3.30	1.65	1.59
ACSS2	acyl-CoA synthetase short-chain family member 2	3.24	1.38	1.55
DHCR7	7-dehydrocholesterol reductase	3.11	1.47	1.48
FDFT1	farnesyl-diphosphate farnesyltransferase 1	2.38	1.24	1.12

**Table 2:** Partial list of genes related to ER stress and autophagy that are upregulated in TAIII-treated cells.

ER stress related transcripts		BT4747	MDAMB231	MCF10A
ATF3	activating transcription factor 3	4.48	13.42	3.30
CTH	cystathionase (cystathionine gamma-lyase)	1.30	8.24	2.83
CTH	cystathionase (cystathionine gamma-lyase)	1.25	6.02	1.61
LRF	chromosome 5 open reading frame 41	6.28	5.44	2.74
CHAC1	ChaC, cation transport regulator homolog 1 (E. coli)	0.74	5.21	1.39
DDIT3	DNA-damage-inducible transcript 3/CHOP	0.81	4.68	1.40
HERPUD1	homocysteine-, endoplasmic reticulum stress-inducible	1.35	4.65	1.25
ASNS	asparagine synthetase	1.09	4.38	2.12
TRIB3	tribbles homolog 3 (Drosophila)	0.71	4.12	1.89
SLC38A2	solute carrier family 38, member 2	1.94	3.20	1.82
SLC2A3	solute carrier family 2 (facilitated glucose transporter), member 3	1.01	3.05	0.70
PPP1R15A	protein phosphatase 1, regulatory (inhibitor) subunit 15A/GADD34	1.46	3.01	3.53
SLC3A2	solute carrier family 3 (activators of dibasic and neutral amino acid transport)	0.95	2.93	2.19
CEBPB	CCAAT/enhancer binding protein (C/EBP), beta	0.73	2.87	0.83
GLS	glutaminase	1.16	2.75	3.21
NDRG1	N-myc downstream regulated gene 1	2.00	2.69	3.65
SLC16A3	solute carrier family 16, member 3 (monocarboxylic acid transporter 4)	0.91	2.41	1.26
XBP1	X-box binding protein 1	0.57	2.33	1.88
SLC1A4	solute carrier family 1 (glutamate/neutral amino acid transporter), member 4	0.73	2.3	3.42
HSPA6	heat shock 70 kDa protein 6 (HSP70B′)	2.53	1.17	2.26
EIF2AK3	eukaryotic translation initiation factor 2-alpha kinase 3/PERK	2.17	1.29	1.76
DNAJB9	DnaJ (Hsp40) homolog, subfamily B, member 9	2.16	1.77	1.23
Autophagy- related transcripts				
ULK1	unc-51-like kinase 1 (C. elegans)	1.61	4.80	3.47
SQSTM1	sequestosome 1	1.87	3.51	1.69
MAP1LC3B	microtubule-associated protein 1 light chain 3 beta	1.89	2.66	1.44

**Figure 3:**
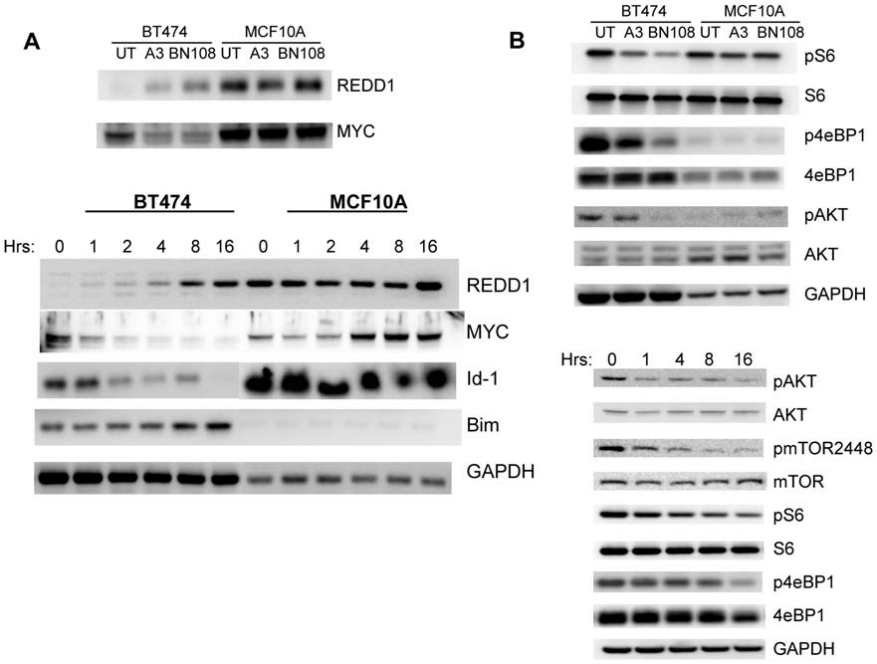
TAIII and BN108 induce similar changes in gene expression and inhibit major proliferative signal transduction pathways selectively in cancer cells. A. Both BN108 and TAIII induce expression of REDD1 and inhibit MYC in BT474 but not in MCF10A cells. Top panels, cell extracts were prepared fro untreated cells and cells treated with BN108 (0.5 mg/ml) and TAIII (2.5 µM in BT474 and 7.5 µM for MCF10A) for 4 hours. Bottom panels, time course of expression of the indicated proteins in BT474 and MCF10A cells treated with TAIII. B. Top, both BN108 and TAIII inhibit phosphorylation of mTORC1 targets s6 ribosomal protein and 4eBP1 as well as AKT kinase selectively in cancer cells. Bottom, time course of inhibition of activating posphorylations of AKT and mTOR, as well as inhibition of phosphorylation of mTORC1 targets s6 and 4eBP1by TAIII in BT474 cells.

In breast cancer cells BN108 and TAIII induced changes in expression of several genes with known functions in apoptosis, which were verified by Western blotting. Expression of REDD1/DDIT4, p21CIP, stratifin and GDF15 proteins was increased during the treatment; while expression of Myc, Id1 and Id3 was decreased (some of these changes are shown in Figure 3A). These proteins were not affected in MCF10A cells. In addition, TAIII induces higher levels of pro-apoptotic protein Bim in BT474 cells but not in MCF10A cells (Figure 3A). Similar changes were observed in MDAMB231 and in prostate carcinoma Du145 (not shown) indicating the possible role of these expression changes in the selective anti-tumor activity of TAIII.

### BN108 and TAIII downregulate the activity of mTORC1

We have examined the potential consequences of the observed increases in levels of REDD1 in BN108 and TAIII treated cancer cells. REDD1/DDIT4 is a stress-responsive gene [22], [23] that has been characterized as a strong inhibitor of mTORC1 pathway [24]–[26]. Indeed, the phosphorylation of mTOR on serine 2448 and phosphorylation of mTORC1 downstream targets 4eBP1 and S6 ribosomal protein were inhibited in BT474 treated with either BN108 or TAIII, as well as in MDAMB231 cells (not shown), but not in MCF10A (Figure 3B).

**Figure 4 pone-0007283-g004:**
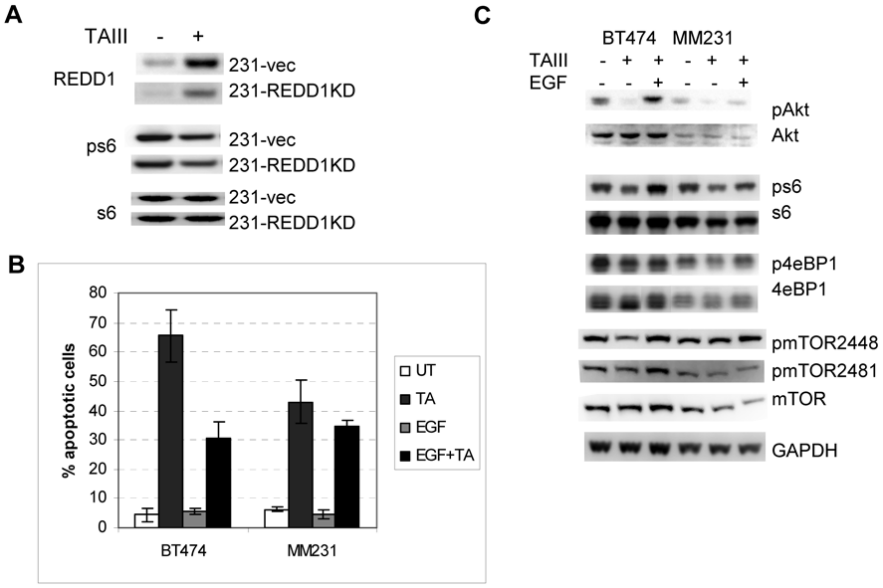
EGF treatment but not silencing of REDD1 provides a partial protective effect against TAIII cytotoxicity. A. Levels of REDD1 and phosphorylation of s6 protein in MDAMB231 cells transduced with control (231-vec) or REDD1 siRNA expressing lentivirus (231-REDD1KD). Cell extracts were analyzed by Western blotting before and 4 hours after treatment with 5 µM of TAIII. B. Effect of addition of exogenous EGF on survival of BT474 and MDAMB231 cells treated with TAIII for 24 hours (4 µM for BT474 and 7.5 µM for MM231). C. Effect of exogenous EGF on Akt activity, mTORC1 activity and mTOR phosphorylation in cells treated with TAIII for 16 hours. Western blotting was done with the phospho-specific antibodies and antibodies detecting total levels of the indicated proteins.

To examine if REDD1 plays a role in the observed inhibition of mTORC1 by TAIII, expression of lentivirus-based siRNA for REDD1 was introduced into MDAMB231 and BT474 cells. REDD1 level in untreated cells was significantly reduced, and induction of REDD1 by TAIII was also adversely affected (Figure 4A). However, neither BT474 nor MDAMB231 cells with reduced REDD1 expression were protected from killing by TAIII (not shown). Moreover, phosphorylation of the downstream targets of mTORC1, 4eBP1 and S6 ribosomal protein was not restored in cells with reduced REDD1 (not shown); indicating that induction of REDD1 might not be the primary cause for the inhibition of mTORC1 activity by TAIII.

Activity of mTORC1 is regulated by many cellular pathways and players other than REDD1, one of the most prominent being the AKT kinase [16], [27]. We have therefore examined if activity of AKT is affected by BN108 and TAIII and found that phosphorylation of AKT is indeed reduced by both treatments in cancer cells but not in MCF10A cells (Figure 4B). mTORC2 phosphorylates the serine 473 of Akt leading to its activation, raising a possibility that activity of mTORC2 is also affected by TAIII.

BT474 cells contain highly amplified and overexpressed HER2 gene, while MDAMB231 have high levels of EGFR that contribute to Akt activation. We then examined if exogenous stimulation of oncogenic receptor tyrosine kinases by a growth factor might sustain the activity of Akt and mTORC1 and confer protection from TAIII. Treatment with TAIII in presence of exogenous EGF indeed conferred a significant protection to BT474 cells and less so to MDAMB231 (Figure 4B). Western blot analysis of cells co-treated with TAIII and EGF revealed that in presence of EGF TAIII fails to elicit a significant reduction in phosphorylation of Akt and mTORC1 targets in BT474 cells (Figure 4C). Examination of the phosphorylation of mTOR itself in cells treated with TAIII in absence or presence of EGF showed that phosphorylation of mTOR on serine 2448 (characteristic of mTOR in mTORC1) and on mTOR on serine 2481 (characteristic of activated mTOR in mTORC2 [28]) are both reduced in TAIII treated BT474 cells but restored in presence of EGF. However, it should be noted that the protection conferred by EGF was only partial, even in BT474 cells where phosphorylation of mTOR, Akt and mTORC1 targets appeared to be effectively sustained in presence of EGF (Figure 4C). This indicates that in addition to the inhibition of mTORC1, TAIII might have other death-promoting effects on tumor cells.

To identify the other potential cellular pathways contributing to TAIII cytotoxicity in tumor cells, we examined the functional role of Id-1 that, along with Id-3 is strongly downregulated in breast cancer cells but not in MCF10A (Figure 3A and Table S2). Exogenous Id-1 was stably expressed in MDAMB231 cells, and higher levels of Id-1 were maintained during TAIII treatment (not shown). However, analysis of Id-1 expressing cells showed that enforced expression of Id-1 levels did not confer an improved survival during TAIII treatment (not shown), indicating that downregulation of Id-1 by TAIII could be consequences of TAIII effects on cell viability or proliferation. Moreover, concomitant downregulation of REDD1 and forced expression of Id-1 did not improve survival of MDAMB231 cells treated with TAIII (not shown).

### TAIII induces ER stress

The results described above show that inhibition of mTORC1 activity during TAIII treatment plays a significant but not exclusive role in TAIII induced apoptosis. We have then turned our attention to the potential significance of the observation that ER stress is induced in cells treated with TAIII, as manifested by expression of ER stress/amino acid starvation/integrated stress response (ISR) markers (Table 2; partial list) and the activation of caspase 4 (Figure 1D). Array expression analyses showed a robust activation in MDAMB231 cells of the known ISR markers, such as CHOP, TRIB3, HEPRUD, ASNS, and solute carriers, while upregulation of some of these was weak or absent in BT474 and MCF10A (Table 2). Interestingly, a gene described as LRF, a negative regulator of unfolded protein response [29], was most strongly induced in BT474 cells.

**Figure 5:**
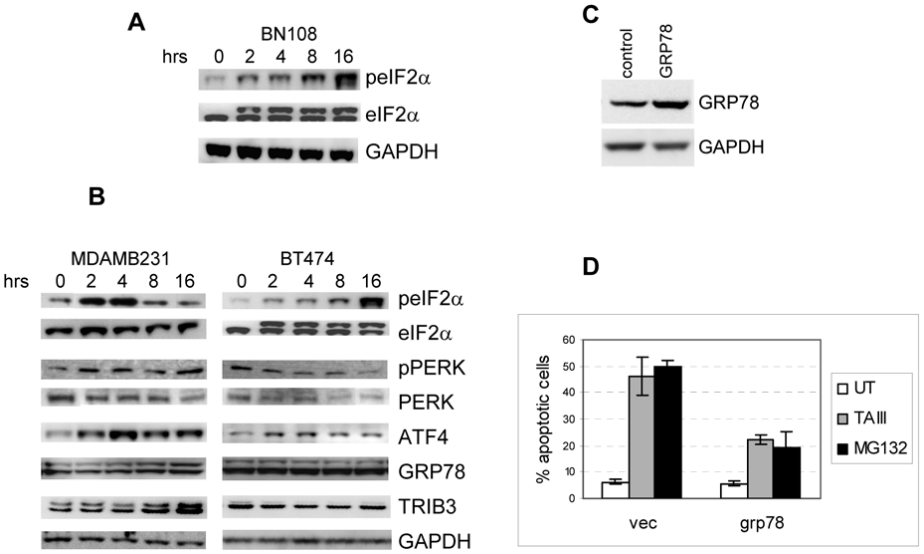
TAIII induces ER stress and protective autophagy. A. Treatment with BN108 induce phosphorylation of translation initiation factor eIF2α. Cell extracts were prepared from BT474 cells treated with BN108 (0.5 mg/ml) for times indicated and subjected to gel electrophoresis and blotting with antibodies against phosphorylated form of eIF2α (Ser51) or total eIF2α protein. B. Western blot analysis of the expression of some markers of ER stress in MDAMB231 and BT474 cells. Cells were treated with TAIII for the indicated times and after electrophoresis extract were immunobloted with indicated antibodies. C. Western blot analysis of MDAMB231 cells transfected with control or GRP78 expressing vector. D. Expression of exogenous GRP78 in MDAMB231 cells offers partial protection against apoptosis induced by TAIII (5 µM) or MG132 (0.25 µM).

In order to verify that breast cancer cells indeed develop ER stress response when treated with BN108 or TAIII, we have examined phosphorylation of the translation initiation factor eIF2α, considered to be a hallmark of ER stress response. We found that eIF2α is phosphorylated in a time–dependent manner in BT474 cells treated with BN108 (Figure 5A) but not in MCF10A (not shown because no appreciable phosphorylation of eIF2α was seen in MCF10A cells either before or after treatment). Similarly, treatment with TAIII also induced phosphorylation of eIF2α in BT474 and MDAMB231 cells even though the maximum phosphorylation was seen in MDAMB231 cells soon after the start of treatment, and in BT474 cells only at 16 hours after treatment start (Figure 5B).

Of the three components of ER stress response, PERK is the only one that phosphorylates eIF2α. We have therefore examined the status of PERK in TAIII-treated cells, and found that PERK is phosphorylated in MDAMB231 cells but not in BT474, in spite of the fact that eIF2α is phosphorylated in both cell lines. Transcription factor ATF4, a major target of induction by activated eIF2α, was strongly induced in MDAMB231 cells and less so in BT474 cells indicating that the processes downstream of eIF2α phosphorylation are intact in both cell lines but are more robust in MDAMB231 cells. The expression array analysis did not show upregulation of RNAs for classical ER stress responders such as PDI, calreticulin and GRP78. To examine if TAIII induces a predominantly translational ER stress response, we conducted a proteomic analysis of MDAMB231 cells treated with TAIII for 4, 8 and 16 hours. PDI and calreticulin protein levels were increased in a time-dependent manner, though the increases were relatively modest (at 16 hours, 77% increase for calreticulin and 70% for PDI; Table S3). We have examined possible changes in levels of GRP78 and other markers of ER stress in breast cancer cells treated with TAIII, An induction in levels of GRP78 and TRIB3 was observed in MDAMB231 cells but not in BT474 cells (Figure 5B), indicating a weak or possibly atypical ER stress response in the latter.

It has been reported that proteolytic activation of the master regulator of cholesterol biosynthesis SREBP-2 [30] and increased expression of its target genes are observed in cells subjected to ER stress [31]–[34]. Expression array analyses indicated upregulation of a number of SREBP-2 target genes in TAIII treated cells (Table 1 and Tables S1 and S2). We have confirmed by Western blot analyses that SREBP-2 is proteolytically activated in cells treated with BN108 or TAIII (Figure S2), and that two of its transcriptional targets isopentenyl-diphosphate delta isomerase (IDI1) and ATP citrate-lyase (ACL), identified in the expression array experiments, are induced in MDAMB231 (not shown), BT474 and MCF10A cells (Figure S2). At the same time, quantification of the cholesterol levels over 24 hours of treatment showed only modest or no changes in cells treated with BN108 or TAIII (Figure S2). This indicates that neither depletion of total cellular cholesterol levels nor cholesterol overload are a likely cause of cell death observed in breast cancer cell lines, in particular because activation of SREBP-2 was also observed in the relatively resistant MCF10A cells (Figure S2). Activation of transcription of INSIG-1, a protein that negatively regulates activation of SREBP-2 [35], [36] could be relevant to the relatively modest increases in the total cholesterol levels in TAIII treated cells (Figure S2). We suggest that TAIII induces ER stress that is strongly manifested in the effects on cholesterol biosynthetic pathway, in particular in BT474 cells, but less so in the activation of the classical markers of ER stress in these cells.

Induction of robust ER stress response can play a protective role and prevent cell death. Chaperon GRP78, when overexpressed, was shown to protect cells from ER stress induced apoptosis [37], [38]. Because induction of GRP78 by TAIII was not observed in BT474 cells and was relatively modest in MDAMB231 cells, we have examined if introduction of GRP78 expression (Figure 5C) could protect the cells from killing by TAIII. Figure 5D shows that expression of GRP78 in MDAMB231 cells indeed conferred a significant degree of protection from apoptosis induced by TAIII, as well as by proteasome inhibitor MG132, a known inducer of ER stress and apoptosis. This supports our hypothesis that, in addition to the mTOR inhibition, ER stress contributes to the TAIII induced apoptosis.

### TAIII induces protective autophagy

Expression array analysis detected induction by TAIII of three transcripts directly related to autophagy: ULK1/ATG1, sequestosome, and MAP1LC3B (Table 2). These data further support the previously published results that TAIII induces autophagy [4]. The TAIII-induced autophagy was shown to play a protective role in TAIII induced death of HeLa cells [4].The possible role of autophagy in cell death induced by TAIII in breast cancer cells was therefore examined.

**Figure 6:**
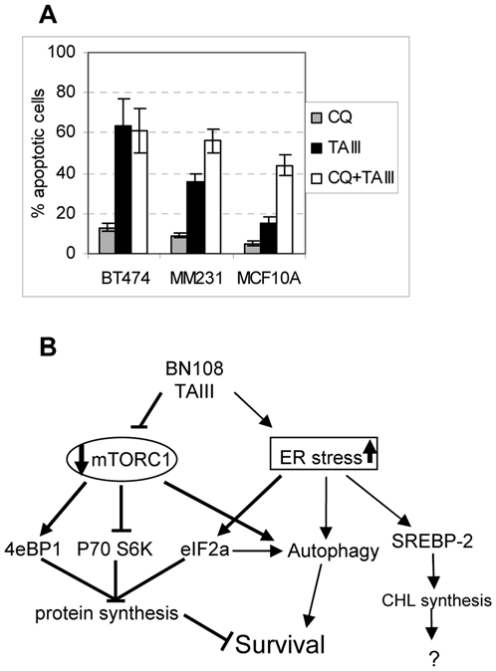
Autophagy induced by TAIII plays a protective role in cell death. A. Inhibition of autophagy augments apoptosis induced by TAIII in MDAMB231 and MCF10A cell lines, but not in BT474. Cells were pretreated for three hours with 20 µM of chloroquine (CQ) after which TAIII (4 µM for BT474, 5 µM for MM231 and 7.5 µM for MCF10A) was added for 24 hours. B. A cartoon illustrating the various cellular effects of BN108 and TAIII. The pathways induced selectively in cancer cells are shown in thicker lines.

Treatment of cells with TAIII in presence of autophagy inhibitor chloroquine revealed that autophagy plays different roles in TAIII induced responses. In the most sensitive cells, BT474, inhibition of autophagy did not affect the outcome of TAIII treatment, indicating that autophagy is either not induced or is not protective in these cells. However, inhibition of autophagy in MDAMB231 and particularly in the MCF10A cells lead to a significantly increased cell death in response to TAIII (Figure 6A). Autophagy also played a protective role in the TAIII induced apoptosis in a breast cancer line SKBr3 and, as previously reported, HeLa cells (not shown). Induction of autophagic responses was confirmed by following levels and distribution of GFP-LC3 in transfected MDAMB231 cells after treatment with TAIII, as well as Western blot analysis of LC3 cleavage (Figure S3). However, treatment of BT474 cells with TAIII has not affected distribution of the transfected GFP-LC3 (not shown) indicating that these cells do not develop autophagy in response to TAIII. We conclude that the previously reported induction of autophagy by TAIII [4] is protective in a variety of cells including the TAIII-resistant cells, and that lack of autophagic response to TAIII is associated with increased sensitivity to its pro-apoptotic effects.

## Discussion

This work describes the selective anti-tumor activity of BN108, an herbal extract used in Traditional Chinese Medicine (TCM), and identifies its major cytotoxic chemical component, TAIII. This conclusion is based on several lines of evidence: TAIII demonstrates the same selectivity towards breast cancer cell lines as BN108; TAIII induces apoptosis involving caspase-4 activation similar to BN108; TAIII induces transcriptional changes in breast cancer cells that largely overlap with these induced by BN108; both TAIII and BN108 inhibit mTORC1 in cancer cells and induce protective autophagy. Another major and more abundant timosaponin from BN108, TBII, could be enzymatically converted to TAIII by the removal of extra sugar, which unmasks its cytotoxicity. Since BN108, like most herbal extracts in TCM, is taken orally, it will be of interest to determine if TBII could be converted to active TAIII *in vivo* by glycosidases produced by intestinal microflora.

Perhaps not surprisingly, we found that both TAIII and BN108 elicit several different cellular responses, some of which were observed in all cells examined, and some that were selectively activated in tumor cells but not in the more resistant normal cells (Figure 6B). The interplay between cellular pathways that are differentially activated or inhibited by TAIII in different cells probably determines the fate of cells subjected to treatment. The strong inhibitory effect of TAIII on activity of mTORC1 was observed selectively in tumor cells. The mTORC1 is frequently inappropriately activated in cancer and is contributing to the transformed phenotype [15] , and this could play a role in the increased sensitivity of certain cancer cells to TAIII . The precise mechanism of mTORC1 inhibition by TAIII remains to be determined, but it involves growth factor activated proliferative pathways (Figure 4B) and does not involve depletion of energy stores (ATP levels are not affected in cells treated with TAIII or BN108; data not shown). Some transcription changes induced by TAIII are indicative of amino acid starvation response (Table 2), that could contribute to inhibition of mTORC1. In any case, selective inhibition of mTORC1 and Akt in cancer cells versus non-transformed cells is very likely to contribute to the selective cytotoxicity of BN108 and TAIII.

mTORC1 is well known to keep autophagy in check [13], and its inhibition could be a culprit in TAIII induced autophagy. However, inhibition of mTORC1 by TAIII in BT474 cells (Figure 3B) did not induce protective autophagy. One could hypothesize that BT474 might simply harbor a defect in a gene whose product plays a role in autophagy, but other cytotoxic treatment do induce autophagic response in BT474 (data not shown). At the same time, protective autophagy was induced by TAIII in MCF10A cells, where mTORC1 was not inhibited. This would indicate that TAIII could induce autophagy through cellular pathways other than mTORC1 inhibition.

Indeed, we have identified a second cellular response elicited by TAIII that could be a potent inducer of autophagy: ER stress, manifested in the induction of a number of ER stress markers, and, importantly, phosphorylation of eIF2α, the central node in the integrated stress responses [5]. No eIF2α phosphorylation was detected in MCF10A cells, even though they show some transcriptional responses associated with ER stress (Table 2) and activate cholesterol biosynthesis pathway (Figure S2).

Phosphorylation of eIF2α could promote a stress-resistant state as part of integrated stress responses (ISR), but could also promote apoptosis. It apparently correlates with apoptosis induction by TAIII in breast cancer cells. In MDAMB231 cells, a much more robust transcriptional ER stress response is seen than in BT474 cells (Table 2) indicating that the ER stress transcriptional program that should follow eIF2α phosphorylation is not completed in BT474. We have examined if salubrinal, a selective inhibitor of eIF2α de-phosphorylation [39], can influence the outcome of TAIII treatment, but salubrinal did not change the status of peIF2α or the extent of apoptosis in any of the cell lines including BT474 (not shown), indicating that dephosphorylation of eIF2α is not responsible for the relative weakness of ER stress responses in BT474.

In turn, the weak ER stress response of BT474 could be causative in the lack of induction of protective autophagy in these cells, and their increased sensitivity to TAIII. In particular, the lack of induction of GRP78 and a weak upregulation of ATF4 (Figure 5) in BT474 could contribute to lack of autophagy since both these ER stress responders were shown to contribute to autophagy [40], [41]. It is also possible that the dual effects of TAIII on mTORC1 and ER make different contributions to induction of apoptosis versus protective autophagy in different cells. Our data also suggest that lack of significant cytotoxic activity of TAIII towards normal cells could be related to two notable differences in their responses compared to cancer cells: first, lack of mTORC1 inhibition; second, a significant role of autophagy in protecting nontransformed cells from TAIII (Figure 5D).

The role of the observed induction of SREBP-2 and its target genes in TAIII cytotoxicity remains to be elucidated, though it is unlikely to be directly relevant to cell death, because SREBP-2 is induced in relatively resistant MCF10A cells. One possibility is that TAIII induces specific changes in ER cholesterol levels rather than large fluctuation in total cellular cholesterol. Even small changes in ER cholesterol content can trigger changes in the SREBP-2 status through activating protein SCAP and inhibitor INSIG-1 [35]. The other intriguing possibility is based on the described antagonism between integrated stress response (ISR) and sterol-regulated gene expression in cells treated with selected ER stress inducing compounds [42]. In MDAMB231 cells the ER stress response is prominent (Table 2 and Figure 5) and SREBP activation is less pronounced (Table 1), while in BT474 markers of ER stress are either not induced or induced weakly (Table 2 and Figure 5), but SREBP-2 activation is very strong (Table 1). Harding et al. described this reciprocal relationship of the ISR and sterol-regulated gene expression and showed its dependence on eIF2α phosphorylation [42]. Perhaps more surprisingly, in this work deletion of PERK, the only known ER stress responsive kinase that phosphorylates eIF2α, did not lead to the abrogation of eIF2α phosphorylation in cells treated with the compounds. We show that even though PERK is not phosphorylated in BT474 cells treated with TAIII, eIF2α is phosphorylated. Therefore, in BT474 cells a different mechanism, not involving PERK, might be responsible for activation of eIF2α. There are recent evidence that ER stress could induce phosphorylation of eIF2α by mechanisms independent of PERK [43].

We have not observed induction of ROS that was recently described in HeLa cells subjected to treatment with TAIII [4]. This difference between our observations and the previously reported results may stem from at least two potential sources: first, it could be related to the different concentrations of TAIII used. In the paper by Sy et al., [4], IC50 of TAIII was close to 10 µM for HeLa cells after two day treatment, whereas the two breast cancer cell lines we have used were significantly more sensitive, with IC50 values of about 2.5 and 6 µM after 1 day. Second, and more important, the relatively modest increase in cellular ROS (about 30–40% over baseline) reported in HeLa cells [4] was observed between 24 to 48 hours of treatment with TAIII. We examined the effects of BN108 and TAIII on mitochondria during the first 4–6 hours of treatment with TAIII or BN108, and could not detect appreciable induction of ROS or dissipation of the mitochondrial membrane potential. At this time, however, inhibition of mTORC1 and phosphorylation of eIF2α (Figures 3–5) were evident, indicating that these changes well precede the mitochondrial events. Mitochondrial degeneration could play a role in the TAIII induced death, but it would most likely occur as consequences of the other, described here, events triggered by TAIII earlier.

It is potentially important for the understanding of the mechanisms of TAIII cytotoxicity that both responses elicited by it in tumor cells, i.e. inhibition of mTORC1 and ER stress, induce autophagy [8], [9], [13]. TAIII induced autophagy is protective in both tumor and normal cells, but the pro-apoptotic effects of the mTORC1 inhibition and ER stress appear to prevail in tumor cells. These results indicate that TAIII could be a promising candidate for drug development as a selective anti-cancer agent.

## Supporting Information

Table S1 Expression changes induced in BT474 cells treated with BN108
(0.07 MB PDF)

Table S2 Expression analysis data of genes affected by TAIII in BT474, MDAMB231 and MCF10A cells
(0.10 MB PDF)

Table S3 Proteomic analysis of MDAMB231 cells treated with TAIII
(0.06 MB PDF)

Methods S1 Expression array analysis
(0.02 MB DOC)

Figure S1 Activation of caspases 4 and 9 by BN108. Extracts prepared fromBT474 treated with BN108 for indicated times were analyzed for caspase-4 and caspase-9 activity using caspase activity kits from BioVisionaccording to manufacturer's instructions. Caspase 4 inhibitor LEVD (EMD Biosciences) was used at 8 µM to ensure the specificity of the assay.
(0.07 MB PDF)

Figure S2 Effects of BN108 and TAIII on cholesterol synthesis. A. BT474 cells were treated for 4 hours with either 2.5 µM TAIII or 0.5 mg/ml of BN108, and extracts were subjected to Western blotting with antibodies to isopentenyl-diphosphate delta isomerase (IDI-1) and ATP-citrate lyase (ACL), both transcriptional targets of SREBP-2 . B. SREBP-2 is activated by BN108 in both cancer and non-transformed cells. Time course of SREBP-2 activation in MDAMB231 and MCF10A cells treated with BN108 (0.5 mg/ml) for the times indicated. C. TAIII increases levels of SREBP-2 target isopentenyl-diphosphate delta isomerase (IDI-1) in both cancer and nontransformed cells. Cells were treated with TAIII at 2.5 µM (BT474) and 5 µM (MCF10A). D. Total cholesterol levels in MDAMB231 and MCF10A cells treated with TAIII or BN108. E. TAIII increases levels of INSIG-1, inhibitor of SREBP-2 activation. BT474 cells were treated with TAIII and extracts blotted with anti-INSIG-1 antibody.
(0.65 MB PDF)

Figure S3 TAIII treatment induces autophagy. MDAMB231 cells were transfected with GFP-LC3 expressing construct and subjected to treatment with 5 µM of TAIII for 16 hours. A. Immunofluorescent detection of EGFP-LC3 in untreated (mock) and treated cells. B. Western blot analysis of the same cells treated with TAIII and detected with an antibody to LC3. Both GFP-LC3-I and proteolytically cleaved EGFP-LC3-II are detected. Endogenous LC3-I is not detected due to its low expression levels or antibody preference for LC3-II.
(0.09 MB PDF)

## References

[1] Kawasaki T, Yamauchi T (1963). Saponins of Timo (Anemarrhenae Rhizoma). Ii. Structure of Timosaponin a-Iii.. Chem Pharm Bull (Tokyo).

[2] Saito S, Nagase S, Ichinose K (1994). New steroidal saponins from the rhizomes of Anemarrhena asphodeloides Bunge (Liliaceae).. Chem Pharm Bull (Tokyo).

[3] Zhang J, Meng Z, Zhang M, Ma D, Xu S (1999). Effect of six steroidal saponins isolated from anemarrhenae rhizoma on platelet aggregation and hemolysis in human blood.. Clin Chim Acta.

[4] Sy LK, Yan SC, Lok CN, Man RY, Che CM (2008). Timosaponin A-III induces autophagy preceding mitochondria-mediated apoptosis in HeLa cancer cells.. Cancer Res.

[5] Mori K (2000). Tripartite management of unfolded proteins in the endoplasmic reticulum.. Cell.

[6] Ron D, Walter P (2007). Signal integration in the endoplasmic reticulum unfolded protein response.. Nat Rev Mol Cell Biol.

[7] Hitomi J, Katayama T, Eguchi Y, Kudo T, Taniguchi M (2004). Involvement of caspase-4 in endoplasmic reticulum stress-induced apoptosis and Abeta-induced cell death.. J Cell Biol.

[8] Hoyer-Hansen M, Jaattela M (2007). Connecting endoplasmic reticulum stress to autophagy by unfolded protein response and calcium.. Cell Death Differ.

[9] Yorimitsu T, Klionsky DJ (2007). Endoplasmic reticulum stress: a new pathway to induce autophagy.. Autophagy.

[10] Gozuacik D, Kimchi A (2007). Autophagy and cell death.. Curr Top Dev Biol.

[11] Kondo Y, Kanzawa T, Sawaya R, Kondo S (2005). The role of autophagy in cancer development and response to therapy.. Nat Rev Cancer.

[12] Levine B, Yuan J (2005). Autophagy in cell death: an innocent convict?. J Clin Invest.

[13] Diaz-Troya S, Perez-Perez ME, Florencio FJ, Crespo JL (2008). The role of TOR in autophagy regulation from yeast to plants and mammals.. Autophagy.

[14] Proud CG (2009). mTORC1 signalling and mRNA translation.. Biochem Soc Trans.

[15] Sabatini DM (2006). mTOR and cancer: insights into a complex relationship.. Nat Rev Cancer.

[16] Sarbassov DD, Ali SM, Sengupta S, Sheen JH, Hsu PP (2006). Prolonged rapamycin treatment inhibits mTORC2 assembly and Akt/PKB.. Mol Cell.

[17] Jacinto E, Loewith R, Schmidt A, Lin S, Ruegg MA (2004). Mammalian TOR complex 2 controls the actin cytoskeleton and is rapamycin insensitive.. Nat Cell Biol.

[18] Sarbassov DD, Ali SM, Kim DH, Guertin DA, Latek RR (2004). Rictor, a novel binding partner of mTOR, defines a rapamycin-insensitive and raptor-independent pathway that regulates the cytoskeleton.. Curr Biol.

[19] Feldman ME, Apsel B, Uotila A, Loewith R, Knight ZA (2009). Active-site inhibitors of mTOR target rapamycin-resistant outputs of mTORC1 and mTORC2.. PLoS Biol.

[20] Thoreen CC, Kang SA, Chang JW, Liu Q, Zhang J (2009). An ATP-competitive mammalian target of rapamycin inhibitor reveals rapamycin-resistant functions of mTORC1.. J Biol Chem.

[21] Shoemaker M, Hamilton B, Dairkee SH, Cohen I, Campbell MJ (2005). In vitro anticancer activity of twelve Chinese medicinal herbs.. Phytother Res.

[22] Ellisen LW, Ramsayer KD, Johannessen CM, Yang A, Beppu H (2002). REDD1, a developmentally regulated transcriptional target of p63 and p53, links p63 to regulation of reactive oxygen species.. Mol Cell.

[23] Shoshani T, Faerman A, Mett I, Zelin E, Tenne T (2002). Identification of a novel hypoxia-inducible factor 1-responsive gene, RTP801, involved in apoptosis.. Mol Cell Biol.

[24] Brugarolas J, Lei K, Hurley RL, Manning BD, Reiling JH (2004). Regulation of mTOR function in response to hypoxia by REDD1 and the TSC1/TSC2 tumor suppressor complex.. Genes Dev.

[25] Corradetti MN, Inoki K, Guan KL (2005). The stress-inducted proteins RTP801 and RTP801L are negative regulators of the mammalian target of rapamycin pathway.. J Biol Chem.

[26] Reiling JH, Hafen E (2004). The hypoxia-induced paralogs Scylla and Charybdis inhibit growth by down-regulating S6K activity upstream of TSC in Drosophila.. Genes Dev.

[27] Skeen JE, Bhaskar PT, Chen CC, Chen WS, Peng XD (2006). Akt deficiency impairs normal cell proliferation and suppresses oncogenesis in a p53-independent and mTORC1-dependent manner.. Cancer Cell.

[28] Copp J, Manning G, Hunter T (2009). TORC-specific phosphorylation of mammalian target of rapamycin (mTOR): phospho-Ser2481 is a marker for intact mTOR signaling complex 2.. Cancer Res.

[29] Audas TE, Li Y, Liang G, Lu R (2008). A novel protein, Luman/CREB3 recruitment factor, inhibits Luman activation of the unfolded protein response.. Mol Cell Biol.

[30] Horton JD, Goldstein JL, Brown MS (2002). SREBPs: transcriptional mediators of lipid homeostasis.. Cold Spring Harb Symp Quant Biol.

[31] Colgan SM, Tang D, Werstuck GH, Austin RC (2007). Endoplasmic reticulum stress causes the activation of sterol regulatory element binding protein-2.. Int J Biochem Cell Biol.

[32] Kovacs WJ, Tape KN, Shackelford JE, Wikander TM, Richards MJ (2008). Peroxisome deficiency causes a complex phenotype due to hepatic SREBP/Insig dysregulation associated with endoplasmic reticulum stress.. J Biol Chem.

[33] Lee JN, Ye J (2004). Proteolytic activation of sterol regulatory element-binding protein induced by cellular stress through depletion of Insig-1.. J Biol Chem.

[34] Werstuck GH, Lentz SR, Dayal S, Hossain GS, Sood SK (2001). Homocysteine-induced endoplasmic reticulum stress causes dysregulation of the cholesterol and triglyceride biosynthetic pathways.. J Clin Invest.

[35] Radhakrishnan A, Goldstein JL, McDonald JG, Brown MS (2008). Switch-like control of SREBP-2 transport triggered by small changes in ER cholesterol: a delicate balance.. Cell Metab.

[36] Yang T, Espenshade PJ, Wright ME, Yabe D, Gong Y (2002). Crucial step in cholesterol homeostasis: sterols promote binding of SCAP to INSIG-1, a membrane protein that facilitates retention of SREBPs in ER.. Cell.

[37] Rao RV, Peel A, Logvinova A, del Rio G, Hermel E (2002). Coupling endoplasmic reticulum stress to the cell death program: role of the ER chaperone GRP78.. FEBS Lett.

[38] Reddy RK, Mao C, Baumeister P, Austin RC, Kaufman RJ (2003). Endoplasmic reticulum chaperone protein GRP78 protects cells from apoptosis induced by topoisomerase inhibitors: role of ATP binding site in suppression of caspase-7 activation.. J Biol Chem.

[39] Boyce M, Bryant KF, Jousse C, Long K, Harding HP (2005). A selective inhibitor of eIF2alpha dephosphorylation protects cells from ER stress.. Science.

[40] Li J, Ni M, Lee B, Barron E, Hinton DR (2008). The unfolded protein response regulator GRP78/BiP is required for endoplasmic reticulum integrity and stress-induced autophagy in mammalian cells.. Cell Death Differ.

[41] Milani M, Rzymski T, Mellor HR, Pike L, Bottini A (2009). The role of ATF4 stabilization and autophagy in resistance of breast cancer cells treated with Bortezomib.. Cancer Res.

[42] Harding HP, Zhang Y, Khersonsky S, Marciniak S, Scheuner D (2005). Bioactive small molecules reveal antagonism between the integrated stress response and sterol-regulated gene expression.. Cell Metab.

[43] Carra S, Brunsting JF, Lambert H, Landry J, Kampinga HH (2009). HspB8 participates in protein quality control by a non-chaperone-like mechanism that requires eIF2{alpha} phosphorylation.. J Biol Chem.

