# Supplementary material for: Timosaponin AIII Is Preferentially Cytotoxic to Tumor Cells through Inhibition of mTOR and Induction of ER Stress
Source: PLoS One. 2009 Sep 30;4(9):e7283. doi: 10.1371/journal.pone.0007283 (PMC2747272; doi:10.1371/journal.pone.0007283)
Supplement: Table S1 — Expression changes induced in BT474 cells treated with BN108 (0.07 MB PDF) [file pone.0007283.s001.pdf]

Table S1 Expression changes induced in BT474 cells treated with BN108

UPREGULATED GENES

ENTREZ ID	ENTREZ GEN	ENTREZ GENE DESCRIPTION	Fold up	GeneBank ID
1545	CYP1B1	cytochrome P450, family 1, subfamily B, polypeptide 1	5.5	NM_000104
55902	ACAS2	acetyl-Coenzyme A synthetase 2 (ADP forming)	5.1	NM_018677
11182	SLC2A6	solute carrier family 2 (facilitated glucose transporter), member 6	4.7	NM_017585
158471	C9orf65	chromosome 9 open reading frame 65	4.0	NM_138818
23175	LPIN1	lipin 1	3.8	NM_145693
1543	CYP1A1	cytochrome P450, family 1, subfamily A, polypeptide 1	3.7	NM_000499
3422	IDI1	isopentenyl-diphosphate delta isomerase	3.7	NM_004508
6307	SC4MOL	sterol-C4-methyl oxidase-like	3.6	NM_006745
3157	HMGCS1	3-hydroxy-3-methylglutaryl-Coenzyme A synthase 1 (soluble)	3.6	BX537620
333929	SNAI3	snail homolog 3 (Drosophila)	3.4	AY203928
255738	PCSK9	proprotein convertase subtilisin/kexin type 9	3.2	NM_174936
2224	FDPS	farnesyl diphosphate synthase (farnesyl pyrophosphate synthetase, dimethylallylt	3.1	AK021828
51478	HSD17B7	hydroxysteroid (17-beta) dehydrogenase 7	3.0	AK022929
9518	GDF15	growth differentiation factor 15	3.0	NM_004864
54785	FLJ20014	hypothetical protein FLJ20014	2.9	NM_017622
2810	SFN	stratifin	2.8	NM_006142
3638	INSIG1	insulin induced gene 1	2.7	NM_005542
81558	LOC81558	C/EBP-induced protein	2.7	NM_030802
339924	LOC339924	hypothetical protein LOC339924	2.7	AK024270
54541	DDIT4	DNA-damage-inducible transcript 4	2.7	NM_019058
1026	CDKN1A	cyclin-dependent kinase inhibitor 1A (p21, Cip1)	2.7	NM_000389
51003	MED31	mediator of RNA polymerase II transcription, subunit 31 homolog (yeast)	2.6	NM_016060
4597	MVD	mevalonate (diphospho) decarboxylase	2.5	NM_002461
256933	NPB	preproneuropeptide B	2.5	BC073815
39	ACAT2	acetyl-Coenzyme A acetyltransferase 2 (acetoacetyl Coenzyme A thiolase)	2.5	NM_005891
4047	LSS	lanosterol synthase (2,3-oxidosqualene-lanosterol cyclase)	2.5	NM_002340
901	CCNG2	cyclin G2	2.5	AK092638
4598	MVK	mevalonate kinase (mevalonic aciduria)	2.4	NM_000431
47	ACLY	ATP citrate lyase	2.4	NM_001096
51478	HSD17B7	hydroxysteroid (17-beta) dehydrogenase 7	2.2	NM_016371
50814	NSDHL	NAD(P) dependent steroid dehydrogenase-like	2.2	NM_015922
80153	FLJ21128	hypothetical protein FLJ21128	2.2	NM_025083
6812	STXBP1	syntaxin binding protein 1	2.2	NM_003165
10020	GNE	glucosamine (UDP-N-acetyl)-2-epimerase/N-acetylmannosamine kinase	2.2	NM_005476
901	CCNG2	cyclin G2	2.2	NM_004354
8353	HIST1H3E	histone 1, H3e	2.2	BG676315
39	ACAT2	acetyl-Coenzyme A acetyltransferase 2 (acetoacetyl Coenzyme A thiolase)	2.1	NM_005891
8218	CLTCL1	clathrin, heavy polypeptide-like 1	2.1	NM_001835
6309	SC5DL	sterol-C5-desaturase (ERG3 delta-5-desaturase homolog, fungal)-like	2.1	NM_006918
25976	TIPARP	TCDD-inducible poly(ADP-ribose) polymerase	2.1	NM_015508
440433	LOC440433	hypothetical gene supported by AK092630	2.1	AL832615

DOWNREGULATED GENES

ENTREZ ID	ENTREZ GEN	ENTREZ GENE DESCRIPTION	Fold down	GeneBank ID
22943	DKK1	dickkopf homolog 1 (Xenopus laevis)	3.5	NM_012242
7057	THBS1	thrombospondin 1	3.0	NM_003246
3491	CYR61	cysteine-rich, angiogenic inducer, 61	2.9	NM_001554
407975	C13orf25	chromosome 13 open reading frame 25	2.5	NM_213723
79642	FLJ23548	hypothetical protein FLJ23548	2.4	NM_024590
285489	FLJ33718	hypothetical protein FLJ33718	2.4	NM_173660
374	AREG	amphiregulin (schwannoma-derived growth factor)	2.3	NM_001657
57795	KIAA1747	hypothetical protein from clone 24828	2.3	NM_021165
4609	MYC	v-myc myelocytomatosis viral oncogene homolog (avian)	2.2	NM_002467
9052	GPCR5A	G protein-coupled receptor, family C, group 5, member A	2.2	NM_003979

10252	SPRY1	sprouty homolog 1, antagonist of FGF signaling (Drosophila)	2.1	NM_005841
6387	CXCL12	chemokine (C-X-C motif) ligand 12 (stromal cell-derived factor 1)	2.1	NM_000609
285958	LOC285958	hypothetical protein LOC285958	2.1	AK096179
374	AREG	amphiregulin (schwannoma-derived growth factor)	2.1	NM_001657
7424	VEGFC	vascular endothelial growth factor C	2.0	NM_005429
79888	FLJ12443	hypothetical protein FLJ12443	2.0	NM_024830
441951	LOC441951	similar to RPE-spondin	2.0	AK124175
6130	RPL7A	ribosomal protein L7a	2.0	BX641050
9334	B4GALT5	UDP-Gal:betaGlcNAc beta 1,4- galactosyltransferase, polypeptide 5	2.0	NM_004776
595	CCND1	cyclin D1 (PRAD1: parathyroid adenomatosis 1)	2.0	NM_053056
390	ARHE	ras homolog gene family, member E	2.0	NM_005168
799	CALCR	calcitonin receptor	2.0	NM_001742
