# Supplementary material for: Timosaponin AIII Is Preferentially Cytotoxic to Tumor Cells through Inhibition of mTOR and Induction of ER Stress
Source: PLoS One. 2009 Sep 30;4(9):e7283. doi: 10.1371/journal.pone.0007283 (PMC2747272; doi:10.1371/journal.pone.0007283)
Supplement: Table S2 — Expression analysis data of genes affected by TAIII in BT474, MDAMB231 and MCF10A cells (0.10 MB PDF) [file pone.0007283.s002.pdf]

Table S2
Expression analysis data of genes affected by TAIII in BT474, MDAMB231 and MCF10A cells

Genes upregulated by TAIII; Ranked by fold change in BT474

TargetID	Accession	GeneDescription	BT474	MDAMB231	MCF10a
RN7SK	NR_001445.1	RNA, 7SK, nuclear	24.27	1.67	0.70
INSIG1	NM_198337.1	insulin induced gene 1	8.71	6.14	1.84
SC4MOL	NM_006745.3	sterol-C4-methyl oxidase-like	7.64	2.07	1.35
LPIN1	NM_145693.1	lipin 1	6.69	2.31	2.22
LOC153222	NM_153607.1	chromosome 5 open reading frame 41	6.28	5.44	2.74
SC4MOL	NM_006745.3	sterol-C4-methyl oxidase-like	6.10	2.08	1.24
IRS2	NM_003749.2	insulin receptor substrate 2	5.86	1.47	1.44
HSD17B7	NM_016371.2	hydroxysteroid (17-beta) dehydrogenase 7	5.10	1.33	1.54
HSD17B7P2	NR_003086.1	hydroxysteroid (17-beta) dehydrogenase 7 pseudogene 2	4.73	0.98	1.10
INSIG1	NM_198336.1	insulin induced gene 1	4.72	4.33	1.70
IDI1	NM_004508.2	isopentenyl-diphosphate delta isomerase 1	4.50	1.80	1.19
ATF3	NM_001674.2	activating transcription factor 3	4.48	13.41	3.30
RNF39	NM_025236.2	ring finger protein 39	4.35	0.92	1.24
KLHL24	NM_017644.3	kelch-like 24 (Drosophila)	4.25	2.37	1.35
HMGCS1	NM_002130.4	3-hydroxy-3-methylglutaryl-Coenzyme A synthase 1 (soluble)	4.23	2.06	1.51
PIM1	NM_002648.2	pim-1 oncogene	4.13	3.57	1.76
MVD	NM_002461.1	mevalonate (diphospho) decarboxylase	3.95	1.20	1.39
GNE	NM_005476.3	glucosamine (UDP-N-acetyl)-2-epimerase/N-acetylmannosamine kinase	3.83	1.49	0.98
CREB5	NM_182899.3	cAMP responsive element binding protein 5	3.81	3.59	1.76
CCDC28A	NM_015439.2	coiled-coil domain containing 28A	3.79	1.64	1.39
HMGCR	NM_000859.1	3-hydroxy-3-methylglutaryl-Coenzyme A reductase	3.64	1.32	1.19
LDLR	NM_000527.2	low density lipoprotein receptor (familial hypercholesterolemia)	3.54	2.37	1.70
SQLE	NM_003129.3	squalene epoxidase	3.32	1.05	1.27
PGM2L1	NM_173582.3	phosphoglucomutase 2-like 1	3.30	1.65	1.59
ACSS2	NM_018677.2	acyl-CoA synthetase short-chain family member 2	3.24	1.38	1.55
KLF2	NM_016270.2	Kruppel-like factor 2 (lung)	3.23	4.95	4.70
HBP1	NM_012257.3	HMG-box transcription factor 1	3.13	2.46	1.43
DHCR7	NM_001360.1	7-dehydrocholesterol reductase	3.11	1.47	1.48
JUN	NM_002228.3	jun oncogene	3.05	1.27	1.23
DUSP1	NM_004417.2	dual specificity phosphatase 1	2.98	3.68	1.86
GRB7	NM_005310.2	growth factor receptor-bound protein 7	2.91	1.12	1.42
SC5DL	NM_001024956	sterol-C5-desaturase (ERG3 delta-5-desaturase homolog, S. cerevisiae)-like	2.91	1.67	1.88
CAMTA1	NM_015215.1	calmodulin binding transcription activator 1	2.85	1.17	0.80
KLF6	NM_001300.4	Kruppel-like factor 6	2.84	3.29	1.78
KLHL28	NM_017658.3	kelch-like 28 (Drosophila)	2.72	2.59	1.60
ABTB1	NM_172027.1	ankyrin repeat and BTB (POZ) domain containing 1	2.72	1.62	2.06
YPEL2	NM_001005404	yippee-like 2 (Drosophila)	2.71	1.67	1.20
MNT	NM_020310.2	MAX binding protein	2.67	2.41	1.56
KLF6	NM_001300.4	Kruppel-like factor 6	2.64	2.56	1.73
ZFYVE1	NM_178441.1	zinc finger, FYVE domain containing 1	2.63	2.71	1.74
ACSS2	NM_018677.2	acyl-CoA synthetase short-chain family member 2	2.63	1.23	1.53
KIAA0831	NM_014924.3	KIAA0831	2.55	1.43	1.13
MGC17330	NM_052880.3	phosphoinositide-3-kinase interacting protein 1	2.54	1.38	1.27
CDKN1A	NM_000389.2	cyclin-dependent kinase inhibitor 1A (p21, Cip1)	2.54	3.86	1.19
FAM117A	NM_030802.2	family with sequence similarity 117, member A	2.53	0.81	0.68
RNF38	NM_194329.2	ring finger protein 38	2.53	1.65	1.58
HSPA6	NM_002155.3	heat shock 70kDa protein 6 (HSP70B')	2.53	1.17	2.26
MEF2D	NM_005920.2	myocyte enhancer factor 2D	2.51	1.84	1.53
PRIC285	NM_033405.2	peroxisomal proliferator-activated receptor A interacting complex 285	2.51	2.44	1.76
NSDHL	NM_015922.1	NAD(P) dependent steroid dehydrogenase-like	2.51	0.82	1.18
ZBTB34	XM_937669.2	zinc finger and BTB domain containing 34	2.49	1.86	1.95
CYP51A1	NM_000786.2	cytochrome P450, family 51, subfamily A, polypeptide 1	2.48	1.09	1.00
SLU7	NM_006425.4	SLU7 splicing factor homolog (S. cerevisiae)	2.48	1.55	1.05
FOXO3A	NM_201559.1	forkhead box O3	2.47	1.71	1.18
GRB7	NM_005310.2	growth factor receptor-bound protein 7	2.47	0.84	1.64
TRIM23	NM_001656.3	tripartite motif-containing 23	2.45	1.36	1.31
TSC1	NM_000368.3	tuberous sclerosis 1	2.43	1.79	1.46
FBXO32	NM_058229.2	F-box protein 32	2.40	1.85	1.45
TRIM4	NM_033017.2	tripartite motif-containing 4	2.39	1.04	0.85
FDFT1	NM_004462.3	farnesyl-diphosphate farnesyltransferase 1	2.38	1.24	1.12
RBM33	NM_001008408	RNA binding motif protein 33	2.38	1.89	1.90
IGF1R	NM_000875.3	insulin-like growth factor 1 receptor	2.36	1.38	1.32
KIF3C	NM_002254.5	kinesin family member 3C	2.33	0.96	0.82
LRCH4	NM_002319.2	leucine-rich repeats and calponin homology (CH) domain containing 4	2.32	1.73	1.95
FOXO3A	NM_001455.2	forkhead box O3	2.32	1.87	1.45
CLK1	NM_004071.2	CDC-like kinase 1	2.31	1.60	1.42
KREMEN2	NM_145348.1	Homo sapiens kringle containing transmembrane protein 2 (KREMEN2), transcr	2.29	0.96	0.87
NPC1	NM_000271.2	Niemann-Pick disease, type C1	2.29	1.37	1.48
WDR19	NM_025132.3	WD repeat domain 19	2.29	1.67	1.96

YPEL1	NM_013313.3	yippee-like 1 (Drosophila)	2.28	1.15	1.17
RHOB	NM_004040.2	ras homolog gene family, member B	2.28	15.34	2.52
ARL6IP6	NM_152522.2	ADP-ribosylation-like factor 6 interacting protein 6	2.27	1.33	1.26
CLCN6	NM_021735.1	chloride channel 6	2.27	1.48	1.66
CD55	NM_000574.2	CD55 molecule, decay accelerating factor for complement (Cromer blood group	2.27	1.63	1.98
GDF15	NM_004864.1	growth differentiation factor 15	2.27	3.14	1.69
GABARAPL1	NM_031412.2	GABA(A) receptor-associated protein like 1	2.26	2.33	2.06
CSTF3	NM_001326.2	cleavage stimulation factor, 3' pre-RNA, subunit 3, 77kDa	2.26	1.51	1.43
C17ORF59	NM_017622.1	chromosome 17 open reading frame 59	2.25	1.12	1.18
CREB5	NM_001011666	cAMP responsive element binding protein 5	2.25	2.26	1.58
BNIPL	NM_138278.2	BCL2/adenovirus E1B 19kD interacting protein like	2.24	1.47	2.06
HSD17B7P2	NR_003086.1	hydroxysteroid (17-beta) dehydrogenase 7 pseudogene 2	2.22	1.04	1.58
CLK1	NM_004071.2	CDC-like kinase 1	2.21	1.16	1.34
KIAA1666	XM_942124.2	RIMS binding protein 3	2.21	1.62	0.76
FRAT1	NM_005479.2	frequently rearranged in advanced T-cell lymphomas	2.20	1.22	1.13
ZBTB10	NM_023929.2	zinc finger and BTB domain containing 10	2.19	1.60	0.93
ACTR6	NM_022496.2	ARP6 actin-related protein 6 homolog (yeast)	2.17	0.98	1.07
LSS	NM_002340.3	lanosterol synthase (2,3-oxidosqualene-lanosterol cyclase)	2.17	1.41	1.52
EIF2AK3	NM_004836.3	eukaryotic translation initiation factor 2-alpha kinase 3	2.17	1.29	1.76
MGC14376	NM_032895.2	chromosome 17 open reading frame 91	2.16	1.88	1.62
DNAJB9	NM_012328.1	DnaJ (Hsp40) homolog, subfamily B, member 9	2.16	1.77	1.23
TP53INP1	NM_033285.2	tumor protein p53 inducible nuclear protein 1	2.16	1.90	2.32
FBXO33	NM_203301.1	F-box protein 33	2.15	1.75	1.06
CCNG2	NM_004354.1	cyclin G2	2.15	2.48	1.39
SCG5	NM_003020.1	secretogranin V (7B2 protein)	2.14	0.68	0.92
RICTOR	NM_152756.3	rapamycin-insensitive companion of mTOR	2.14	1.79	1.61
CALCOCO1	NM_020898.1	calcium binding and coiled-coil domain 1	2.14	0.92	0.96
CLDND1	NM_019895.2	claudin domain containing 1	2.14	1.22	1.18
JMJD1A	NM_018433.3	jumonji domain containing 1A	2.14	1.85	1.51
SIRT1	NM_012238.3	sirtuin (silent mating type information regulation 2 homolog) 1 (S. cerevisiae)	2.13	1.55	1.30
ALS2CR13	NM_173511.2	amyotrophic lateral sclerosis 2 (juvenile) chromosome region, candidate 13	2.13	1.18	1.07
ZFP36	NM_003407.1	zinc finger protein 36, C3H type, homolog (mouse)	2.11	2.65	1.56
ANG	NM_001145.2	angiogenin, ribonuclease, RNase A family, 5	2.11	2.04	1.52
FDPS	NM_002004.2	farnesyl diphosphate synthase (farnesyl pyrophosphate synthetase, dimethylally	2.11	0.88	1.15
TMEM55B	NM_144568.1	transmembrane protein 55B	2.11	1.25	1.49
PPP1R10	NM_002714.2	protein phosphatase 1, regulatory (inhibitor) subunit 10	2.10	0.82	0.97
MICA	NM_000247.1	MHC class I polypeptide-related sequence A	2.10	1.20	1.19
MXD4	NM_006454.2	MAX dimerization protein 4	2.10	2.57	1.54
KIAA1683	NM_025249.1	KIAA1683	2.09	1.10	0.82
THRAP6	NM_080651.1	mediator complex subunit 30	2.09	1.07	0.96
ZFYVE1	NM_021260.1	zinc finger, FYVE domain containing 1	2.08	2.43	1.36
ACAT2	NM_005891.1	acetyl-Coenzyme A acetyltransferase 2 (acetoacetyl Coenzyme A thiolase)	2.07	1.01	1.01
ANKRD12	NM_015208.2	ankyrin repeat domain 12	2.07	1.61	1.32
CIR	NM_004882.3	CBF1 interacting corepressor	2.07	1.20	0.85
TULP3	NM_003324.3	tubby like protein 3	2.07	1.30	1.00
WISP3	NM_198239.1	WNT1 inducible signaling pathway protein 3	2.07	0.84	1.26
MVK	NM_000431.1	mevalonate kinase (mevalonic aciduria)	2.06	1.68	1.46
APAF1	NM_181861.1	apoptotic peptidase activating factor 1	2.05	1.75	1.26
DNAJB6	NM_058246.3	DnaJ (Hsp40) homolog, subfamily B, member 6	2.05	1.17	1.41
DUSP10	NM_007207.3	dual specificity phosphatase 10	2.05	1.08	1.37
HS.222909	AL117578	chromosome 21 open reading frame 30	2.04	0.99	1.04
KLF11	XM_938887.1	PREDICTED: Homo sapiens Kruppel-like factor 11 (KLF11), mRNA.	2.04	1.65	1.39
JUND	NM_005354.3	jun D proto-oncogene	2.03	4.42	1.76
TBC1D15	NM_022771.3	TBC1 domain family, member 15	2.03	1.22	1.29
CENPQ	NM_018132.3	centromere protein Q	2.03	1.24	1.13
KATNAL1	NM_001014380	katanin p60 subunit A-like 1	2.02	1.11	1.19
FLJ39575	NM_182597.1	chromosome 7 open reading frame 53	2.02	1.34	2.18
IRF6	NM_006147.2	interferon regulatory factor 6	2.02	1.21	0.92
WDR20	NM_181291.1	WD repeat domain 20	2.02	1.50	1.68
MORC3	NM_015358.1	MORC family CW-type zinc finger 3	2.01	1.35	1.14
C8ORF61	NM_001034061	ceroid-lipofuscinosis, neuronal 8 (epilepsy, progressive with mental retardation)	2.01	1.15	2.00
DSCR1	NM_203418.1	regulator of calcineurin 1	2.01	2.04	0.76
KLHL21	NM_014851.2	kelch-like 21 (Drosophila)	2.01	1.58	1.32
YPEL5	NM_016061.1	yippee-like 5 (Drosophila)	2.01	1.89	1.77
ITLN2	NM_080878.2	intelectin 2	2.01	0.87	0.87
TIPARP	NM_015508.2	TCDD-inducible poly(ADP-ribose) polymerase	2.00	1.00	0.78
NDRG1	NM_006096.2	N-myc downstream regulated gene 1	2.00	2.69	3.65
RRAGC	NM_022157.2	Ras-related GTP binding C	2.00	2.53	1.20

Genes downregulated by TAIII; ranked based on BT474

TargetID	Accession	GeneDescription	BT474	MDAMB231	MCF10A
JUB	NM_032876.4	jub, ajuba homolog (Xenopus laevis)	0.40	0.49	0.92
LMCD1	NM_014583.2	LIM and cysteine-rich domains 1	0.40	0.95	0.79
RASL11B	NM_023940.2	RAS-like, family 11, member B	0.40	0.94	0.99

RERG	NM_032918.1	RAS-like, estrogen-regulated, growth inhibitor	0.40	1.12	0.74
DUSP5	NM_004419.3	dual specificity phosphatase 5	0.39	1.64	1.13
DUSP6	NM_001946.2	dual specificity phosphatase 6	0.39	0.73	0.73
ZNF703	XM_001129663	zinc finger protein 703	0.39	0.54	1.30
C9ORF3	NM_032823.3	chromosome 9 open reading frame 3	0.39	0.64	1.06
CALML5	NM_017422.3	calmodulin-like 5	0.39	0.91	0.80
BAMBI	NM_012342.2	BMP and activin membrane-bound inhibitor homolog (Xenopus laevis)	0.39	1.18	1.07
PUNC	NM_004884.2	putative neuronal cell adhesion molecule	0.39	0.85	0.85
MYC	NM_002467.3	v-myc myelocytomatosis viral oncogene homolog (avian)	0.38	0.97	0.85
DLX3	NM_005220.2	distal-less homeobox 3	0.38	1.07	0.84
ARID5B	NM_032199.1	AT rich interactive domain 5B (MRF1-like)	0.38	1.68	1.46
HS.374257	BX537506	hypothetical protein LOC286167	0.38	0.92	0.67
DUSP4	NM_001394.5	dual specificity phosphatase 4	0.38	0.57	1.04
ST3GAL1	NM_173344.1	ST3 beta-galactoside alpha-2,3-sialyltransferase 1	0.38	0.95	0.81
SFRS7	NM_001031684	splicing factor, arginine/serine-rich 7, 35kDa	0.38	0.59	0.66
HS.25892	AK096179	chromosome 7 open reading frame 40	0.38	0.62	0.90
CITED4	NM_133467.2	Cbp/p300-interacting transactivator, with Glu/Asp-rich carboxy-terminal domain,	0.37	0.96	0.51
SPRED1	NM_152594.1	sprouty-related, EVH1 domain containing 1	0.36	0.96	1.27
LOC145837	XM_934770.1	PREDICTED: Homo sapiens hypothetical protein LOC145837, transcript variant	0.36	0.62	1.01
CXCR4	NM_003467.2	chemokine (C-X-C motif) receptor 4	0.36	0.84	2.94
SMAD6	NM_005585.3	SMAD family member 6	0.36	0.55	1.02
ATOH8	NM_032827.3	atonal homolog 8 (Drosophila)	0.35	0.72	0.83
C8ORF72	NM_147189.2	family with sequence similarity 110, member B	0.35	0.98	0.92
THBS1	NM_003246.2	thrombospondin 1	0.34	0.83	0.60
ENC1	NM_003633.1	ectodermal-neural cortex (with BTB-like domain)	0.34	0.49	0.85
IER3	NM_003897.3	immediate early response 3	0.34	1.38	0.82
RRS1	NM_015169.3	RRS1 ribosome biogenesis regulator homolog (S. cerevisiae)	0.34	0.31	0.45
SLC16A6	NM_004694.2	solute carrier family 16, member 6 (monocarboxylic acid transporter 7)	0.34	0.57	0.72
ID1	NM_181353.1	inhibitor of DNA binding 1, dominant negative helix-loop-helix protein	0.33	0.25	0.90
EDARADD	NM_080738.2	EDAR-associated death domain	0.32	0.92	1.13
MICALCL	NM_032867.2	MICAL C-terminal like	0.32	0.89	1.08
ASCL2	NM_005170.2	achaete-scute complex homolog 2 (Drosophila)	0.32	1.18	1.11
DUSP4	NM_001394.5	dual specificity phosphatase 4	0.31	0.74	0.78
EPN3	NM_017957.1	epsin 3	0.30	0.87	0.67
CCND1	NM_053056.2	cyclin D1	0.30	0.67	0.85
ALDH1A3	NM_000693.1	aldehyde dehydrogenase 1 family, member A3	0.30	0.96	0.76
KIAA0644	NM_014817.3	KIAA0644 gene product	0.30	0.94	1.16
STC2	NM_003714.2	stanniocalcin 2	0.29	1.42	2.91
TMEM46	NM_001007538	transmembrane protein 46	0.27	1.12	0.74
SLITRK6	NM_032229.2	SLIT and NTRK-like family, member 6	0.26	1.04	0.83
SPRY1	NM_199327.1	sprouty homolog 1, antagonist of FGF signaling (Drosophila)	0.26	0.39	1.10
RND3	NM_005168.3	Rho family GTPase 3	0.26	0.89	1.35
IGFBP3	NM_000598.4	insulin-like growth factor binding protein 3	0.24	0.87	0.78
DKFZP761P0	XM_937796.2	homolog of rat pragma of Rnd2	0.22	0.62	1.06
GATA3	NM_001002295	GATA binding protein 3	0.22	0.47	0.75
PPP1R3C	NM_005398.3	protein phosphatase 1, regulatory (inhibitor) subunit 3C	0.21	1.41	1.27
DKK1	NM_012242.2	dickkopf homolog 1 (Xenopus laevis)	0.16	0.40	0.76

Genes upregulated by TAIII; ranked based on MDAMB231

TargetID	Accession	GeneDescription	MDAMB231	BT474	MCF10A
RHOB	NM_004040.2	ras homolog gene family, member B	15.34	2.28	2.52
ATF3	NM_001674.2	activating transcription factor 3	13.42	4.48	3.30
TSC22D3	NM_004089.3	TSC22 domain family, member 3	11.16	1.13	1.58
DDIT4	NM_019058.2	DNA-damage-inducible transcript 4	9.64	1.04	1.30
CTH	NM_001902.4	cystathionase (cystathionine gamma-lyase)	8.24	1.30	2.83
INSIG1	NM_198337.1	insulin induced gene 1	6.14	8.71	1.84
CTH	NM_001902.4	cystathionase (cystathionine gamma-lyase)	6.02	1.25	1.61
LOC153222	NM_153607.1	chromosome 5 open reading frame 41 LRF	5.44	6.28	2.74
KLF9	NM_001206.2	Kruppel-like factor 9	5.30	1.63	1.86
CHAC1	NM_024111.2	ChaC, cation transport regulator homolog 1 (E. coli)	5.21	0.74	1.39
KLF2	NM_016270.2	Kruppel-like factor 2 (lung)	4.95	3.22	4.70
ADM2	NM_024866.4	adrenomedullin 2	4.92	0.69	1.68
ULK1	XM_942125.1	PREDICTED: Homo sapiens unc-51-like kinase 1 (C. elegans) (ULK1), mRNA.	4.85	1.49	3.57
ULK1	NM_003565.1	unc-51-like kinase 1 (C. elegans)	4.80	1.61	3.47
DDIT3	NM_004083.4	DNA-damage-inducible transcript 3	4.68	0.81	1.40
HERPUD1	NM_001010989	homocysteine-inducible, endoplasmic reticulum stress-inducible, ubiquitin-like do	4.65	1.35	1.25
JUND	NM_005354.3	jun D proto-oncogene	4.42	2.03	1.76
ASNS	NM_133436.1	asparagine synthetase	4.38	1.09	2.12
INSIG1	NM_198336.1	insulin induced gene 1	4.34	4.72	1.70
TRIB3	NM_021158.3	tribbles homolog 3 (Drosophila)	4.12	0.71	1.89
SESN2	NM_031459.3	sestrin 2	3.88	0.81	1.71
CDKN1A	NM_000389.2	cyclin-dependent kinase inhibitor 1A (p21, Cip1)	3.86	2.54	1.19
DUSP1	NM_004417.2	dual specificity phosphatase 1	3.68	2.98	1.86

CREB5	NM_182899.3	cAMP responsive element binding protein 5	3.59	3.81	1.76
PIM1	NM_002648.2	pim-1 oncogene	3.57	4.13	1.76
SQSTM1	NM_003900.3	sequestosome 1	3.51	1.87	1.69
KLF6	NM_001300.4	Kruppel-like factor 6	3.29	2.84	1.78
SLC38A2	NM_018976.3	solute carrier family 38, member 2	3.20	1.94	1.82
BHLHB2	NM_003670.1	basic helix-loop-helix domain containing, class B, 2	3.15	0.98	1.82
GDF15	NM_004864.1	growth differentiation factor 15	3.14	2.27	1.69
ZBTB43	NM_014007.2	zinc finger and BTB domain containing 43	3.13	1.59	1.79
SLC2A3	NM_006931.1	solute carrier family 2 (facilitated glucose transporter), member 3	3.05	1.01	0.70
PPP1R15A	NM_014330.2	protein phosphatase 1, regulatory (inhibitor) subunit 15A	3.01	1.46	3.53
LOC387763	XM_941665.2	hypothetical LOC387763	2.97	1.06	1.20
BTG1	NM_001731.1	B-cell translocation gene 1, anti-proliferative	2.97	1.90	1.26
SLC3A2	NM_001013251	solute carrier family 3 (activators of dibasic and neutral amino acid transport), m	2.93	0.95	2.19
PHGDH	NM_006623.2	phosphoglycerate dehydrogenase	2.89	0.99	1.66
CEBPB	NM_005194.2	CCAAT/enhancer binding protein (C/EBP), beta	2.87	0.73	0.83
TNFRSF10D	NM_003840.3	tumor necrosis factor receptor superfamily, member 10d, decoy with truncated c	2.79	1.11	2.03
GLS	NM_014905.2	glutaminase	2.75	1.16	3.21
NFIL3	NM_005384.2	nuclear factor, interleukin 3 regulated	2.73	1.06	1.30
ZFYVE1	NM_178441.1	zinc finger, FYVE domain containing 1	2.71	2.63	1.74
NDRG1	NM_006096.2	N-myc downstream regulated gene 1	2.69	2.00	3.65
KIAA1370	NM_019600.1	KIAA1370	2.69	1.77	2.21
HOXB9	NM_024017.4	homeobox B9	2.67	0.73	1.10
MAP1LC3B	NM_022818.3	microtubule-associated protein 1 light chain 3 beta	2.66	1.89	1.44
DUSP8	NM_004420.1	dual specificity phosphatase 8	2.66	1.98	1.78
ZFP36	NM_003407.1	zinc finger protein 36, C3H type, homolog (mouse)	2.65	2.11	1.56
LOC88523	NM_033111.2	phosphonoformate immuno-associated protein 5	2.64	1.83	3.40
ABL2	NM_005158.2	v-abl Abelson murine leukemia viral oncogene homolog 2 (arg, Abelson-related	2.60	1.01	2.02
KLHL28	NM_017658.3	kelch-like 28 (Drosophila)	2.59	2.72	1.60
ANKRD37	NM_181726.1	ankyrin repeat domain 37	2.57	1.22	2.75
MXD1	NM_002357.2	MAX dimerization protein 1	2.57	1.91	1.85
MXD4	NM_006454.2	MAX dimerization protein 4	2.57	2.10	1.54
KLF6	NM_001300.4	Kruppel-like factor 6	2.56	2.64	1.73
FAM100B	NM_182565.2	family with sequence similarity 100, member B	2.56	1.60	1.70
RRAGC	NM_022157.2	Ras-related GTP binding C	2.53	2.00	1.20
CCNG2	NM_004354.1	cyclin G2	2.48	2.15	1.39
HBP1	NM_012257.3	HMG-box transcription factor 1	2.46	3.13	1.43
TAF15	NM_139215.1	TAF15 RNA polymerase II, TATA box binding protein (TBP)-associated factor, 6	2.45	1.14	1.36
PRIC285	NM_033405.2	peroxisomal proliferator-activated receptor A interacting complex 285	2.44	2.51	1.76
ZFYVE1	NM_021260.1	zinc finger, FYVE domain containing 1	2.43	2.08	1.36
GPT2	NM_133443.1	glutamic pyruvate transaminase (alanine aminotransferase) 2	2.43	0.83	1.94
ZCCHC14	NM_015144.2	zinc finger, CCHC domain containing 14	2.43	1.29	1.71
MNT	NM_020310.2	MAX binding protein	2.41	2.67	1.56
SLC16A3	NM_001042423	solute carrier family 16, member 3 (monocarboxylic acid transporter 4)	2.41	0.91	1.26
MIDN	NM_177401.4	midnolin	2.39	0.61	1.44
CCL20	NM_004591.1	chemokine (C-C motif) ligand 20	2.39	1.12	0.36
GADD45A	NM_001924.2	growth arrest and DNA-damage-inducible, alpha	2.37	0.69	1.09
SERTAD1	NM_013376.1	SERTA domain containing 1	2.37	0.94	1.55
LDLR	NM_000527.2	low density lipoprotein receptor (familial hypercholesterolemia)	2.37	3.54	1.70
KLHL24	NM_017644.3	kelch-like 24 (Drosophila)	2.37	4.25	1.35
GABARAPL1	NM_031412.2	GABA(A) receptor-associated protein like 1	2.33	2.26	2.06
ARL4A	NM_005738.3	ADP-ribosylation factor-like 4A	2.33	0.72	1.17
EGR1	NM_001964.2	early growth response 1	2.33	0.98	1.06
XBP1	NM_005080.2	X-box binding protein 1	2.33	0.57	1.88
RNF103	NM_005667.2	ring finger protein 103	2.33	1.61	2.07
LPIN1	NM_145693.1	lipin 1	2.32	6.69	2.22
SCD	NM_005063.4	stearoyl-CoA desaturase (delta-9-desaturase)	2.31	1.30	1.07
SLC1A4	NM_003038.2	solute carrier family 1 (glutamate/neutral amino acid transporter), member 4	2.30	0.73	3.42
C16ORF72	NM_014117.2	chromosome 16 open reading frame 72	2.29	1.66	1.63
CEBPG	NM_001806.2	CCAAT/enhancer binding protein (C/EBP), gamma	2.28	1.14	1.32
LOC1000085	NR_003287.1	28S ribosomal RNA	2.28	3.38	0.83
CREB5	NM_001011666	cAMP responsive element binding protein 5	2.26	2.25	1.58
CDKN1A	NM_078467.1	cyclin-dependent kinase inhibitor 1A (p21, Cip1)	2.24	1.09	1.09
LOC221442	NM_001010871	Homo sapiens hypothetical protein LOC221442 (LOC221442), mRNA.	2.24	1.64	1.90
MKNK2	NM_199054.1	MAP kinase interacting serine/threonine kinase 2	2.24	1.38	1.16
CTSK	NM_000396.2	cathepsin K	2.23	1.89	2.34
FOS	NM_005252.2	v-fos FBJ murine osteosarcoma viral oncogene homolog	2.22	1.15	0.74
TES	NM_015641.2	testis derived transcript (3 LIM domains)	2.22	1.05	1.34
HNRPDL	NR_003249.1	heterogeneous nuclear ribonucleoprotein D-like	2.21	1.37	1.34
LARP6	NM_018357.2	La ribonucleoprotein domain family, member 6	2.20	0.94	1.05
PSPH	NM_004577.3	phosphoserine phosphatase	2.20	0.77	1.70
FNIP1	NM_001008738	folliculin interacting protein 1	2.18	1.82	1.45
PNPLA8	NM_015723.2	patatin-like phospholipase domain containing 8	2.18	1.80	1.25
TMEM159	NM_020422.3	transmembrane protein 159	2.18	0.92	1.43
CCND2	NM_001759.2	cyclin D2	2.15	0.99	1.08
C1ORF63	NM_207035.1	Homo sapiens chromosome 1 open reading frame 63 (C1orf63), transcript varia	2.15	1.94	1.90

FAM107B	NM_031453.2	family with sequence similarity 107, member B	2.14	1.02	1.49
CBX4	NM_003655.2	chromobox homolog 4 (Pc class homolog, Drosophila)	2.13	0.87	1.65
PCK2	NM_004563.2	phosphoenolpyruvate carboxykinase 2 (mitochondrial)	2.13	1.08	1.88
CELSR3	NM_001407.1	cadherin, EGF LAG seven-pass G-type receptor 3 (flamingo homolog, Drosophi	2.12	1.47	1.28
MAFG	NM_032711.2	v-maf musculoaponeurotic fibrosarcoma oncogene homolog G (avian)	2.11	1.52	2.50
PSAT1	NM_021154.3	phosphoserine aminotransferase 1	2.10	0.93	1.38
ABL2	NM_007314.1	v-abl Abelson murine leukemia viral oncogene homolog 2 (arg, Abelson-related	2.09	0.96	1.01
SC4MOL	NM_006745.3	sterol-C4-methyl oxidase-like	2.08	6.10	1.24
SLC6A9	NM_001024845	solute carrier family 6 (neurotransmitter transporter, glycine), member 9	2.08	1.02	2.41
SC4MOL	NM_006745.3	sterol-C4-methyl oxidase-like	2.07	7.64	1.35
NXF1	NM_001081491	nuclear RNA export factor 1	2.07	1.43	1.91
HMGCS1	NM_002130.4	3-hydroxy-3-methylglutaryl-Coenzyme A synthase 1 (soluble)	2.07	4.23	1.51
RRAD	NM_004165.1	Ras-related associated with diabetes	2.06	0.94	1.03
TRIB1	NM_025195.2	tribbles homolog 1 (Drosophila)	2.06	1.03	1.05
TES	NM_152829.1	testis derived transcript (3 LIM domains)	2.06	1.14	1.21
OTUD1	XM_001134465	OTU domain containing 1	2.06	1.88	1.64
FAM53C	NM_016605.1	family with sequence similarity 53, member C	2.06	1.74	1.41
DSCR1	NM_203418.1	regulator of calcineurin 1	2.04	2.01	0.76
ANG	NM_001145.2	angiogenin, ribonuclease, RNase A family, 5	2.04	2.11	1.52
KIAA0323	NM_015299.2	KIAA0323	2.04	1.75	1.77
RAB33B	NM_031296.1	RAB33B, member RAS oncogene family	2.03	1.76	1.07
VEGFA	NM_001025366	vascular endothelial growth factor A	2.03	0.96	2.29
ZDHHC11	NM_024786.1	zinc finger, DHHC-type containing 11	2.03	1.18	2.92
DKFZP451A2	NM_001003399	DKFZp451A211 protein	2.03	1.22	1.35
RPS6KA5	NM_004755.2	ribosomal protein S6 kinase, 90kDa, polypeptide 5	2.02	1.54	1.53
TUFT1	NM_020127.1	tuftelin 1	2.02	1.60	1.39
ZSWIM6	XM_936273.2	zinc finger, SWIM-type containing 6	2.02	1.56	2.00
DIDO1	NM_080797.2	death inducer-obliterator 1	2.02	1.46	1.92
TMEM154	NM_152680.1	transmembrane protein 154	2.01	1.16	1.27
ARNTL	NM_001178.4	aryl hydrocarbon receptor nuclear translocator-like	2.00	1.42	1.19

Genes downregulated by TAIII; ranked based on MDAMB231

TargetID	Accession	GeneDescription	MDAMB231	BT474	MCF10A
DKK1	NM_012242.2	dickkopf homolog 1 (Xenopus laevis)	0.40	0.16	0.76
IRS1	NM_005544.1	insulin receptor substrate 1	0.40	0.53	0.64
SPRY1	NM_199327.1	sprouty homolog 1, antagonist of FGF signaling (Drosophila)	0.39	0.26	1.10
RCL1	NM_005772.2	RNA terminal phosphate cyclase-like 1	0.39	0.61	0.53
TMEM177	NM_030577.1	transmembrane protein 177	0.39	0.61	0.73
PRR3	NM_025263.2	proline rich 3	0.38	0.59	0.85
SPRY1	NM_199327.1	sprouty homolog 1, antagonist of FGF signaling (Drosophila)	0.38	0.43	0.87
LOC728285	XM_001127070	similar to keratin associated protein 2-4	0.38	1.04	0.98
SHROOM2	NM_001649.2	shroom family member 2	0.38	0.86	0.72
ALDH1B1	NM_000692.3	aldehyde dehydrogenase 1 family, member B1	0.37	0.78	0.50
ZNF239	NM_005674.1	zinc finger protein 239	0.36	0.87	0.58
PRMT6	NM_018137.1	protein arginine methyltransferase 6	0.36	0.44	0.55
MMACHC	NM_015506.1	methylmalonic aciduria (cobalamin deficiency) cblC type, with homocystinuria	0.36	0.45	0.56
HAS2	NM_005328.1	hyaluronan synthase 2	0.34	0.74	1.05
ZNF485	NM_145312.2	zinc finger protein 485	0.33	0.47	0.55
FJX1	NM_014344.2	four jointed box 1 (Drosophila)	0.33	0.50	0.65
NOG	NM_005450.2	noggin	0.33	0.75	0.84
NDP	NM_000266.1	Norrie disease (pseudoglioma)	0.32	0.96	0.95
RRS1	NM_015169.3	RRS1 ribosome biogenesis regulator homolog (S. cerevisiae)	0.31	0.34	0.45
RIN2	NM_018993.2	Ras and Rab interactor 2	0.30	0.79	0.63
BMP4	NM_130850.1	bone morphogenetic protein 4	0.28	1.05	0.77
BDNF	NM_001709.3	brain-derived neurotrophic factor	0.26	1.09	0.89
ID1	NM_181353.1	inhibitor of DNA binding 1, dominant negative helix-loop-helix protein	0.25	0.33	0.90
RGS4	NM_005613.3	regulator of G-protein signaling 4	0.25	0.99	0.97
ID3	NM_002167.2	inhibitor of DNA binding 3, dominant negative helix-loop-helix protein	0.13	0.69	0.79

Genes upregulated by TAIII; ranked based on MCF10A

TargetID	Accession	GeneDescription	MCF10A	BT474	MDAMB231
RASIP1	NM_017805.2	Ras interacting protein 1	7.08	1.09	1.06
KLF2	NM_016270.2	Kruppel-like factor 2 (lung)	4.70	3.22	4.95
NDRG1	NM_006096.2	N-myc downstream regulated gene 1	3.65	2.00	2.69
ULK1	XM_942125.1	PREDICTED: Homo sapiens unc-51-like kinase 1 (C. elegans) (ULK1), mRNA.	3.57	1.49	4.85
PPP1R15A	NM_014330.2	protein phosphatase 1, regulatory (inhibitor) subunit 15A	3.53	1.46	3.01
ULK1	NM_003565.1	unc-51-like kinase 1 (C. elegans)	3.47	1.61	4.80
SLC1A4	NM_003038.2	solute carrier family 1 (glutamate/neutral amino acid transporter), member 4	3.42	0.73	2.30
LOC88523	NM_033111.2	phosphonoformate immuno-associated protein 5	3.40	1.83	2.64
CLDN15	NM_014343.1	claudin 15	3.38	1.79	1.58

LOC728715	XM_001128260	similar to cDNA sequence BC048546	3.32	1.63	1.68
ATF3	NM_001674.2	activating transcription factor 3	3.30	4.48	13.41
GLS	NM_014905.2	glutaminase	3.21	1.16	2.75
MFNG	NM_002405.2	MFNG O-fucosylpeptide 3-beta-N-acetylglucosaminyltransferase	2.95	1.11	1.14
CXCR4	NM_003467.2	chemokine (C-X-C motif) receptor 4	2.94	0.36	0.84
ZDHHC11	NM_024786.1	zinc finger, DHHC-type containing 11	2.92	1.18	2.03
CXCR4	NM_003467.2	chemokine (C-X-C motif) receptor 4	2.91	0.46	1.28
STC2	NM_003714.2	stanniocalcin 2	2.91	0.29	1.42
CTH	NM_001902.4	cystathionase (cystathionine gamma-lyase)	2.83	1.30	8.24
ANKRD37	NM_181726.1	ankyrin repeat domain 37	2.75	1.22	2.57
LOC153222	NM_153607.1	chromosome 5 open reading frame 41	2.74	6.28	5.44
P2RY11	NM_002566.4	purinergic receptor P2Y, G-protein coupled, 11	2.73	1.70	0.93
C7ORF41	NM_152793.2	chromosome 7 open reading frame 41	2.66	1.32	1.67
MC1R	NM_002386.2	melanocortin 1 receptor (alpha melanocyte stimulating hormone receptor)	2.60	1.43	1.60
RHOB	NM_004040.2	ras homolog gene family, member B	2.52	2.28	15.34
AMH	NM_000479.2	anti-Mullerian hormone	2.51	1.16	1.68
CD7	NM_006137.6	CD7 molecule	2.51	1.49	1.02
MAFG	NM_032711.2	v-maf musculoaponeurotic fibrosarcoma oncogene homolog G (avian)	2.50	1.52	2.11
EPB41L5	NM_020909.2	erythrocyte membrane protein band 4.1 like 5	2.46	1.55	1.49
LAMB3	NM_000228.2	laminin, beta 3	2.46	0.78	1.87
RHBDL1	NM_003961.1	rhomboid, veinlet-like 1 (Drosophila)	2.43	1.65	1.20
SLC6A9	NM_001024845	solute carrier family 6 (neurotransmitter transporter, glycine), member 9	2.41	1.02	2.08
LYST	NM_000081.2	lysosomal trafficking regulator	2.40	1.50	1.77
CTSK	NM_000396.2	cathepsin K	2.34	1.89	2.23
SLC7A11	NM_014331.3	solute carrier family 7, (cationic amino acid transporter, y+ system) member 11	2.34	1.01	1.99
EIF5	NM_001969.3	eukaryotic translation initiation factor 5	2.33	1.21	1.31
TP53INP1	NM_033285.2	tumor protein p53 inducible nuclear protein 1	2.32	2.16	1.90
ERBB3	NM_001982.2	v-erb-b2 erythroblastic leukemia viral oncogene homolog 3 (avian)	2.31	1.59	1.12
VEGFA	NM_001025366	vascular endothelial growth factor A	2.29	0.96	2.03
SLC45A4	XM_933796.2	solute carrier family 45, member 4	2.27	1.63	1.58
CECR7	XM_927968.1	cat eye syndrome chromosome region, candidate 7	2.26	1.25	1.18
HSPA6	NM_002155.3	heat shock 70kDa protein 6 (HSP70B')	2.26	2.53	1.17
PFKFB4	NM_004567.2	6-phosphofructo-2-kinase/fructose-2,6-biphosphatase 4	2.25	1.40	1.79
KIAA0182	NM_014615.1	KIAA0182	2.24	0.91	1.40
LPIN1	NM_145693.1	lipin 1	2.22	6.69	2.31
ZNF398	NM_170686.1	zinc finger protein 398	2.21	1.51	1.30
MGC16121	XM_001128419	hypothetical protein MGC16121	2.21	0.93	1.68
KIAA1370	NM_019600.1	KIAA1370	2.21	1.77	2.69
LTB4R	NM_181657.1	leukotriene B4 receptor	2.20	1.58	1.47
LOC653103	XM_926036.1	PREDICTED: Homo sapiens similar to Ankyrin repeat domain protein 11 (Ankyr	2.20	1.21	1.94
ADSSL1	NM_152328.3	adenylosuccinate synthase like 1	2.19	0.81	1.16
SLC3A2	NM_001013251	solute carrier family 3 (activators of dibasic and neutral amino acid transport), m	2.19	0.95	2.93
FLJ39575	NM_182597.1	chromosome 7 open reading frame 53	2.18	2.02	1.34
FLJ46906	XM_928441.1	hypothetical gene supported by AK128874; BC071813	2.18	0.92	1.22
ADAM32	NM_145004.4	ADAM metallopeptidase domain 32	2.17	0.85	0.94
BSDC1	NM_018045.4	BSD domain containing 1	2.17	1.70	1.72
SPOCK2	NM_014767.1	sparc/osteonectin, cwcv and kazal-like domains proteoglycan (testican) 2	2.17	1.24	1.34
DNHD1	NM_144666.1	dynein heavy chain domain 1	2.16	1.86	1.44
ZXDC	NM_025112.4	ZXD family zinc finger C	2.15	1.02	1.51
CBS	NM_000071.1	cystathionine-beta-synthase	2.15	0.87	0.97
AXUD1	NM_033027.2	AXIN1 up-regulated 1	2.14	1.24	1.90
KIAA1641	XM_944061.1	PREDICTED: Homo sapiens KIAA1641, transcript variant 7 (KIAA1641), mRNA	2.14	1.25	1.53
PLAC8L1	NM_001029869	PLAC8-like 1	2.13	1.26	1.38
AMY2A	NM_000699.2	amylase, alpha 2A (pancreatic)	2.12	1.63	0.93
CCDC64	NM_207311.2	coiled-coil domain containing 64	2.12	1.15	1.50
ASNS	NM_133436.1	asparagine synthetase	2.12	1.09	4.38
MACF1	NM_012090.3	microtubule-actin crosslinking factor 1	2.11	1.03	1.26
C9ORF24	NM_032596.3	chromosome 9 open reading frame 24	2.09	1.31	1.27
RNF103	NM_005667.2	ring finger protein 103	2.07	1.61	2.33
VAMP1	NM_016830.2	vesicle-associated membrane protein 1 (synaptobrevin 1)	2.07	1.00	1.42
SPSB3	NM_080861.3	splA/ryanodine receptor domain and SOCS box containing 3	2.06	1.59	1.88
GABARAPL1	NM_031412.2	GABA(A) receptor-associated protein like 1	2.06	2.26	2.33
ABTB1	NM_172027.1	ankyrin repeat and BTB (POZ) domain containing 1	2.06	2.72	1.62
LOC440345	XM_933717.1	PREDICTED: Homo sapiens hypothetical protein LOC440345, transcript variant	2.06	1.30	1.38
BNIPL	NM_138278.2	BCL2/adenovirus E1B 19kD interacting protein like	2.06	2.24	1.47
PNPLA7	NM_152286.2	patatin-like phospholipase domain containing 7	2.06	1.08	1.07
ARID3B	NM_006465.2	AT rich interactive domain 3B (BRIGHT-like)	2.04	1.46	1.88
GSDML	NM_001042471	gasdermin-like	2.04	1.91	1.15
TNFRSF10D	NM_003840.3	tumor necrosis factor receptor superfamily, member 10d, decoy with truncated c	2.03	1.11	2.79
ABL2	NM_005158.2	v-abl Abelson murine leukemia viral oncogene homolog 2 (arg, Abelson-related	2.02	1.01	2.60
COX19	NM_001031617	COX19 cytochrome c oxidase assembly homolog (S. cerevisiae)	2.02	1.08	1.87
C16ORF79	NM_182563.2	chromosome 16 open reading frame 79	2.02	1.83	1.10
SLC25A34	NM_207348.1	solute carrier family 25, member 34	2.02	1.60	1.48
ATXN1	NM_000332.2	ataxin 1	2.01	1.08	1.73
CHRNA10	NM_020402.2	cholinergic receptor, nicotinic, alpha 10	2.01	1.02	1.49

RNF24	NM_007219.2	ring finger protein 24	2.00	0.84	1.48
C8ORF61	NM_001034061	ceroid-lipofuscinosis, neuronal 8 (epilepsy, progressive with mental retardation)	2.00	2.01	1.15
ZSWIM6	XM_936273.2	zinc finger, SWIM-type containing 6	2.00	1.56	2.02
ATXN2L	XM_939199.1	PREDICTED: Homo sapiens ataxin 2-like, transcript variant 1 (ATXN2L), mRNA	2.00	0.95	1.67
C6ORF128	NM_145316.2	chromosome 6 open reading frame 128	2.00	1.24	1.90

Genes downregulated by TAIII; ranked based on MCF10A

TargetID	Accession	GeneDescription	MCF10A	BT474	MDAMB231
CHST4	NM_005769.1	carbohydrate (N-acetylglucosamine 6-O) sulfotransferase 4	0.40	0.93	0.48
CMAH	NR_002174.1	cytidine monophosphate-N-acetylneuraminic acid hydroxylase (CMP-N-acetylne	0.39	0.65	0.79
FST	NM_013409.1	follistatin	0.38	1.02	0.48
MMP9	NM_004994.2	matrix metallopeptidase 9 (gelatinase B, 92kDa gelatinase, 92kDa type IV collag	0.38	0.88	0.97
TNF	NM_000594.2	tumor necrosis factor (TNF superfamily, member 2)	0.37	1.12	0.91
CCL20	NM_004591.1	chemokine (C-C motif) ligand 20	0.36	1.12	2.39
CSF2	NM_000758.2	colony stimulating factor 2 (granulocyte-macrophage)	0.34	0.92	0.92
CXCL2	NM_002089.1	chemokine (C-X-C motif) ligand 2	0.34	0.77	1.42
IL7R	NM_002185.2	interleukin 7 receptor	0.33	0.96	0.61
CXCR7	NM_020311.2	chemokine (C-X-C motif) receptor 7	0.33	0.52	0.80
IL7R	XM_001127146	interleukin 7 receptor	0.32	1.22	0.66
ZBED2	NM_024508.3	zinc finger, BED-type containing 2	0.32	0.97	0.99
PTX3	NM_002852.2	pentraxin-related gene, rapidly induced by IL-1 beta	0.32	1.00	0.55
CDH11	NM_001797.2	cadherin 11, type 2, OB-cadherin (osteoblast)	0.31	0.96	0.88
IL1A	NM_000575.3	interleukin 1, alpha	0.26	0.91	1.08
IL8	NM_000584.2	interleukin 8	0.24	1.20	1.58
CXCL1	NM_001511.1	chemokine (C-X-C motif) ligand 1 (melanoma growth stimulating activity, alpha)	0.22	1.20	0.75
