# Supplementary material for: Timosaponin AIII Is Preferentially Cytotoxic to Tumor Cells through Inhibition of mTOR and Induction of ER Stress
Source: PLoS One. 2009 Sep 30;4(9):e7283. doi: 10.1371/journal.pone.0007283 (PMC2747272; doi:10.1371/journal.pone.0007283)
Supplement: Table S3 — Proteomic analysis of MDAMB231 cells treated with TAIII (0.06 MB PDF) [file pone.0007283.s003.pdf]

Table S3. Proteomic analysis of MDAMB231 cells treated with TAIII for 4, 8 and 16 hours

	Fold change in abundance over untreated					
Score	4hrs	8 hrs	16 hrs		Accession	Gene description
28	1.90	2.43	2.75	HUMAN	P48643	T-complex protein 1 subunit epsilon OS=Homo sapiens GN=CCT5 PE=1 SV=1
25	1.21	1.64	2.28	HUMAN	Q9NX09	DNA-damage-inducible transcript 4 protein OS=Homo sapiens GN=REDD-1 PE=1 SV=2
27	1.37	1.80	2.03	HUMAN	P62937	Peptidyl-prolyl cis-trans isomerase A OS=Homo sapiens GN=PPIA PE=1 SV=2
19	1.65	1.88	1.90	HUMAN	Q01105	Protein SET OS=Homo sapiens GN=SET PE=1 SV=3
43	1.30	1.54	1.81	HUMAN	Q07021	Complement component 1 Q subcomponent-binding protein, mitochondrial OS=Homo sapiens GN=C1QBP PE=1 SV=1
33	1.44	1.63	1.77	RABIT	P15253	Calreticulin OS=Oryctolagus cuniculus GN=CALR PE=1 SV=1
39	1.27	1.60	1.72	HUMAN	P07737	Profilin-1 OS=Homo sapiens GN=PFN1 PE=1 SV=2
47	1.87	1.81	1.71	BOVIN	Q5E9B7	Chloride intracellular channel protein 1 OS=Bos taurus GN=CLIC1 PE=2 SV=3
57	1.38	1.64	1.70	HUMAN	P07237	Protein disulfide-isomerase OS=Homo sapiens GN=P4HB PE=1 SV=3
118	1.15	1.21	1.22	BOVIN	P63103	14-3-3 protein zeta/delta OS=Bos taurus GN=YWHAZ PE=1 SV=1
21	1.06	1.25	1.24	HUMAN	O14737	Programmed cell death protein 5 OS=Homo sapiens GN=PDCD5 PE=1 SV=3
42	0.95	0.86	0.56	HUMAN	P30041	Peroxiredoxin-6 OS=Homo sapiens GN=PRDX6 PE=1 SV=3
23	0.71	0.53	0.35	HUMAN	P41134	DNA-binding protein inhibitor ID-1 OS=Homo sapiens GN=ID-1 PE=1 SV=1
22	0.63	0.59	0.34	PIG	P54613	Serine/threonine-protein phosphatase 2A 65 kDa regulatory subunit A beta isoform OS=Homo sapiens GN=PPP2R1B PE=1 SV=3

Only proteins with scores above 20, and whose changes in expression were sustained overtime are shown.
