# Supplementary material for: Timosaponin AIII Is Preferentially Cytotoxic to Tumor Cells through Inhibition of mTOR and Induction of ER Stress
Source: PLoS One. 2009 Sep 30;4(9):e7283. doi: 10.1371/journal.pone.0007283 (PMC2747272; doi:10.1371/journal.pone.0007283)
Supplement: Methods S1 — Expression array analysis (0.02 MB DOC) [file pone.0007283.s004.doc]

Supplementary Methods

**RNA isolation and microarray analysis**

Total cellular RNA was isolated utilizing the Aurum RNA isolation kit, Bio-Rad (Hercules, CA), per the manufacturer’s manual. RNAs were first quantified by standard spectrophotometry, and then qualitatively evaluated by capillary electrophoresis employing the Bio-Rad Experion system per the manufacturers’ instruction.

Biotin labeled aRNA samples for hybridization against Illumina Human, V.2, BeadChip microarrays were prepared using the Ambion Illumina TotalPrep RNA Amplification Kit (Applied Biosystems, Foster City, CA). Following synthesis and purification, the biotin-labeled aRNA samples were evaluated by both spectrophotometry and capillary electrophoresis. Subsequent microarray hybridization was performed per the standard Illumina protocol via the University of California San Francisco Genomic Core Facility.
