# Supplementary material for: Timosaponin AIII Is Preferentially Cytotoxic to Tumor Cells through Inhibition of mTOR and Induction of ER Stress
Source: PLoS One. 2009 Sep 30;4(9):e7283. doi: 10.1371/journal.pone.0007283 (PMC2747272; doi:10.1371/journal.pone.0007283)
Supplement: Figure S1 — Activation of caspases 4 and 9 by BN108. Extracts prepared fromBT474 treated with BN108 for indicated times were analyzed for caspase-4 and caspase-9 activity using caspase activity kits from BioVisionaccording to manufacturer's instructions. Caspase 4 inhibitor LEVD (EMD Biosciences) was used at 8 µM to ensure the specificity of the assay. (0.07 MB PDF) [file pone.0007283.s005.pdf]

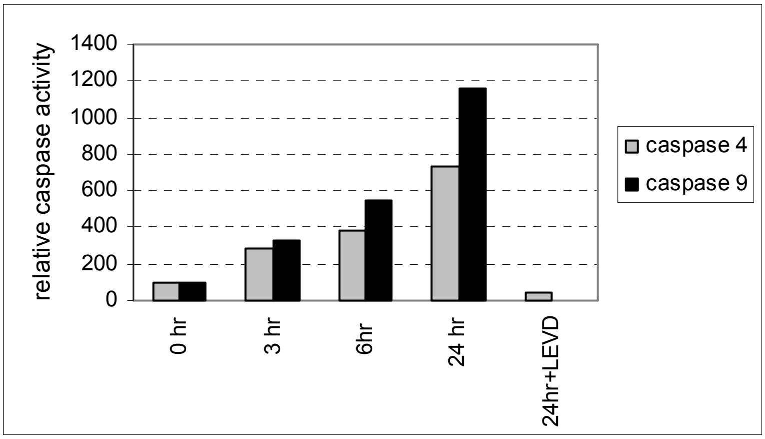

Figure S1. Activation of caspases 4 and 9 by BN108. Extracts prepared from BT474 treated with BN108 for indicated times were analyzed for caspase-4 and caspase-9 activity using caspase activity kits from BioVision according to manufacturer's instructions. Caspase 4 inhibitor LEVD (EMD Biosciences) was used at 8 μM to ensure the specificity of the assay.
