# Supplementary material for: Timosaponin AIII Is Preferentially Cytotoxic to Tumor Cells through Inhibition of mTOR and Induction of ER Stress
Source: PLoS One. 2009 Sep 30;4(9):e7283. doi: 10.1371/journal.pone.0007283 (PMC2747272; doi:10.1371/journal.pone.0007283)
Supplement: Figure S2 — Effects of BN108 and TAIII on cholesterol synthesis. A. BT474 cells were treated for 4 hours with either 2.5 µM TAIII or 0.5 mg/ml of BN108, and extracts were subjected to Western blotting with antibodies to isopentenyl-diphosphate delta isomerase (IDI-1) and ATP-citrate lyase (ACL), both transcriptional targets of SREBP-2 . B. SREBP-2 is activated by BN108 in both cancer and non-transformed cells. Time course of SREBP-2 activation in MDAMB231 and MCF10A cells treated with BN108 (0.5 mg/ml) for the times indicated. C. TAIII increases levels of SREBP-2 target isopentenyl-diphosphate delta isomerase (IDI-1) in both cancer and nontransformed cells. Cells were treated with TAIII at 2.5 µM (BT474) and 5 µM (MCF10A). D. Total cholesterol levels in MDAMB231 and MCF10A cells treated with TAIII or BN108. E. TAIII increases levels of INSIG-1, inhibitor of SREBP-2 activation. BT474 cells were treated with TAIII and extracts blotted with anti-INSIG-1 antibody. (0.65 MB PDF) [file pone.0007283.s006.pdf]

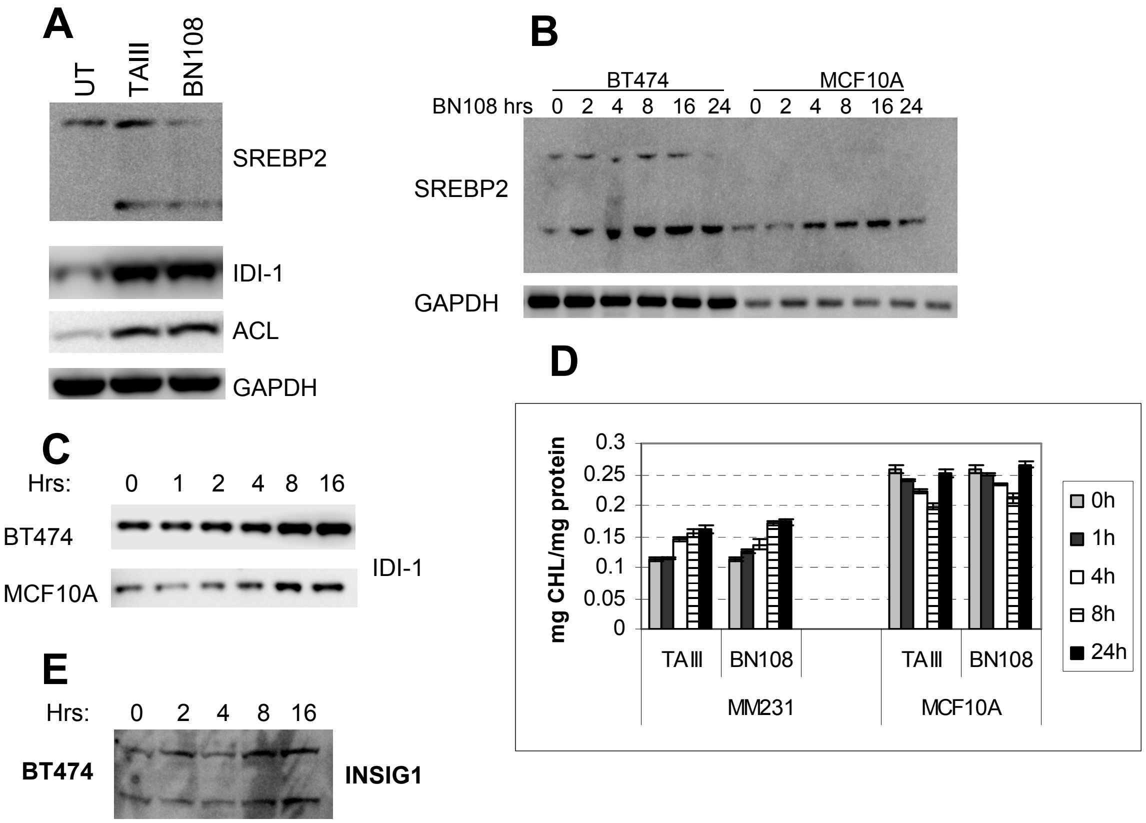

Figure S2 Effects of BN108 and TAIII on cholesterol synthesis. **A**. BT474 cells were treated for 4 hours with either 2.5 μM TAIII or 0.5 mg/ml of BN108, and extracts were subjected to Western blotting with antibodies to isopentenyl-diphosphate delta isomerase (IDI-1) and ATP-citrate lyase (ACL), both transcriptional targets of SREBP-2 . **B**. SREBP-2 is activated by BN108 in both cancer and non-transformed cells. Time course of SREBP-2 activation in MDAMB231 and MCF10A cells treated with BN108 (0.5 mg/ml) for the times indicated. **C**. TAIII increases levels of SREBP-2 target isopentenyl-diphosphate delta isomerase (IDI-1) in both cancer and nontransformed cells. Cells were treated with TAIII at 2.5μM (BT474) and 5 μM (MCF10A). **D**. Total cholesterol levels in MDAMB231 and MCF10A cells treated with TAIII or BN108. **E**. TAIII increases levels of INSIG-1, inhibitor of SREBP-2 activation. BT474 cells were treated with TAIII and extracts blotted with anti-INSIG-1 antibody.
