# Supplementary material for: Timosaponin AIII Is Preferentially Cytotoxic to Tumor Cells through Inhibition of mTOR and Induction of ER Stress
Source: PLoS One. 2009 Sep 30;4(9):e7283. doi: 10.1371/journal.pone.0007283 (PMC2747272; doi:10.1371/journal.pone.0007283)
Supplement: Figure S3 — TAIII treatment induces autophagy. MDAMB231 cells were transfected with GFP-LC3 expressing construct and subjected to treatment with 5 µM of TAIII for 16 hours. A. Immunofluorescent detection of EGFP-LC3 in untreated (mock) and treated cells. B. Western blot analysis of the same cells treated with TAIII and detected with an antibody to LC3. Both GFP-LC3-I and proteolytically cleaved EGFP-LC3-II are detected. Endogenous LC3-I is not detected due to its low expression levels or antibody preference for LC3-II. (0.09 MB PDF) [file pone.0007283.s007.pdf]

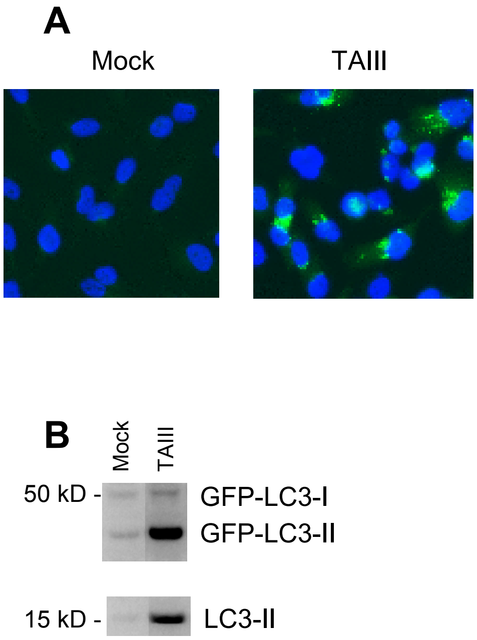

Figure S3. TAIII treatment induces autophagy. MDAMB231 cells were transfected with GFP-LC3 expressing construct and subjected to treatment with 5 μM of TAIII for 16 hours. **A.** Immunofluorescent detection of EGFP-LC3 in untreated (mock) and treated cells. **B.** Western blot analysis of the same cells treated with TAIII and detected with an antibody to LC3. Both GFP-LC3-I and proteolytically cleaved EGFP-LC3-II are detected. Endogenous LC3-I is not detected due to its low expression levels or antibody preference for LC3-II.
